# A novel stemness-hypoxia-related signature for prognostic stratification and immunotherapy response in hepatocellular carcinoma

**DOI:** 10.1186/s12885-022-10195-1

**Published:** 2022-10-28

**Authors:** Genhao Zhang, Kai Zhang, Yanteng Zhao, Qiankun Yang, Xianping Lv

**Affiliations:** 1 Department of Blood Transfusion, the First Affiliated Hospital of Zhengzhou University, Zhengzhou, China; 2 Department of Medical Laboratory, Zhengzhou University Third Affiliated Hospital, Zhengzhou, China

**Keywords:** HCC, Prognosis, Hypoxia, Stemness, TME

## Abstract

**Background:**

The specific differentiation potential, unlimited proliferation, and self-renewal capacity of cancer stem cells (CSCs) are closely related to the occurrence, recurrence, and drug resistance of hepatocellular carcinoma (HCC), as well as hypoxia. Therefore, an in-depth analysis of the relationship between HCC stemness, oxygenation status, and the effectiveness of immunotherapy is necessary to improve the poor prognosis of HCC patients.

**Methods:**

The weighted gene co-expression network analysis (WGCNA) was utilized to find hypoxia-related genes, and the stemness index (mRNAsi) was evaluated using the one-class logistic regression (OCLR) technique. Based on stemness-hypoxia-related genes (SHRGs), population subgroup categorization using NMF cluster analysis was carried out. The relationship between SHRGs and survival outcomes was determined using univariate Cox regression. The LASSO-Cox regression strategy was performed to investigate the quality and establish the classifier associated with prognosis. The main effect of risk scores on the tumor microenvironment (TME) and its response to immune checkpoint drugs was also examined. Finally, qRT-PCR was performed to explore the expression and prognostic value of the signature in clinical samples.

**Results:**

After identifying tumor stemness- and hypoxia-related genes through a series of bioinformatics analyses, we constructed a prognostic stratification model based on these SHRGs, which can be effectively applied to the prognostic classification of HCC patients and the prediction of immune checkpoint inhibitors (ICIs) efficacy. Independent validation of the model in the ICGC cohort yielded good results. In addition, we also constructed hypoxic cell models in Herp3B and Huh7 cells to verify the expression of genes in the prognostic model and found that C7, CLEC1B, and CXCL6 were not only related to the tumor stemness but also related to hypoxia. Finally, we found that the constructed signature had a good prognostic value in the clinical sample.

**Conclusions:**

We constructed and validated a stemness-hypoxia-related prognostic signature that can be used to predict the efficacy of ICIs therapy. We also verified that C7, CLEC1B, and CXCL6 are indeed associated with stemness and hypoxia through a hypoxic cell model, which may provide new ideas for individualized immunotherapy.

**Supplementary Information:**

The online version contains supplementary material available at 10.1186/s12885-022-10195-1.

## Introduction

A small number of specialized cells called cancer stem cells (CSCs) have been reported to be present in hepatocellular carcinoma (HCC) tissue, which has a strong tumorigenic capacity and stem cell properties, including the ability to self-renew and differentiate [1]. CSCs are significantly associated with the poor prognosis of HCC patients through their involvement in the regulation of tumor metastasis, recurrence, and resistance to chemotherapy and radiotherapy for clinical treatment [2, 3]. Moreover, considering the presence of anti-apoptotic and maintenance of stemness components in the tumor microenvironment (TME), CSCs can facilitate the conversion of non-CSCs to CSCs by modifying the critical signaling pathways of normal stem cells in concert with TME, further affecting the poor prognosis of HCC patients [4, 5]. Hypoxia is a common phenotype of liver cancer and increases the risk of immune evasion and recurrence of tumor cells [6]. The hypoxic microenvironment induced by hypoxia is closely related to CSCs [7]. Hypoxia-inducible factor (HIF) secreted by immune cells is a key factor for cancer cell survival and promotes the persistence of cancer stem cells in a hypoxic microenvironment [8–10]. By focusing on several pathways, including the Wnt/-catenin, Notch, and STAT3 pathways, hypoxia can modify the stemness of CSCs, making tumor cells in the G1/S phase resistant to radiation [10, 11]. Given that it is still impossible to predict cancer stemness and oxygenation status based on clinicopathological data from patients, there is an urgent need for validated molecular biomarkers to evaluate tumor cell stemness and oxygenation status. This is necessary for a thorough understanding and eradication of CSCs and hypoxia status, improving the poor survival outcomes of patients and the efficacy of anti-tumor therapy in HCC.

In the present study, through a series of methodical bioinformatic studies, we merged tumor cell stemness and hypoxic state in HCC and created a unique prognostic signature that can forecast patients' response to immunosuppressive medication. In addition, we also constructed a hypoxic cell model to verify the expression of genes in the prognostic model. Therefore, we suggest that this classifier can be used in clinical work as a molecular diagnostic assay to assess the prognostic risk and therapeutic effect of HCC patients.

## Methods

### Publicly available datasets

RNA sequencing (RNA-seq) data from The Cancer Genome Atlas-HCC (TCGA-HCC) cohort obtained from the UCSC Xena project (https://xenabrowser.net) served as the discovery cohort. RNA-seq data of the HCC (LIRI-JP) cohort obtained from the ICGC database (https://dcc.icgc.org/) served as the test cohort. Samples without complete survival data or with a survival time of less than 1 month were excluded. RNA-seq data of the normal samples from the GTEx database obtained from the UCSC Xena project (https://xenabrowser.net) served as a supplementary cohort. The clinical features of the datasets were shown in Table S1. The transcriptome data were normalized using the log2 (FPKM + 1) transformation. Combat from the R package "SVA" was used to rectify the batch effects between the normalized data from the TCGA and GTEx. With cut-off criteria of *P*-value less than 0.05 and |logFC|≥ 1, differentially expressed genes (DEGs) between normal and HCC samples in the TCGA and GTEx cohorts were eliminated.

### Calculation of the mRNAsi and identification of mRNAsi-related DEGs

One-class logistic regression (OCLR) algorithm was used to assess the stemness index (mRNAsi) in normal and HCC samples [12]. Kaplan–Meier Plotter was used to compare differences in survival between patients with high and low mRNAsi. The mRNAsi-related DEGs were identified between patients with high and low mRNAsi with cut-off criteria of *P*-value less than 0.05 and |logFC|≥ 1.

### Calculation of the hypoxia signature score and identification of hypoxia-related genes

Hypoxia signature score was assessed by the ssGSEA method based on the gene set of HALLMARK_HYPOXIA and hypoxia-related genes were then identified by the weighted gene co-expression network analysis (WGCNA) [13]. Gene significance (GS) was also employed to quantify the relationships between specific genes and the hypoxic signature score, while module members represented the relationships between specific genes and gene expression patterns for each module. The *P*-value threshold of GS less than 0.0001 and the significance level of univariate Cox regression with a *P*-value less than 0.01 were used to assess genes found from the module that was most related to the hypoxia signature score as candidates.

### Identification of population subgroups by the non-negative matrix factorization (NMF) algorithm

Overlapping genes between DEGs, mRNAsi-related DEGs, and hypoxia-related genes were considered as stemness- and hypoxia-related genes (SHRGs) for NMF cluster analysis with the criterion "brunet" and 50 iterations. The optimal number of clusters was explored based on cophenetic, dispersion, and profile, as we previously reported [14]. Further Kaplan–Meier survival analysis was done to compare the survival rates of various subtypes found by the NMF algorithm.

### Prognostic risk score model construction

To determine the relationship between SHRGs and patient survival outcomes, univariate Cox regression was used. The prognostic SHRGs were then investigated to create a classifier linked to prognosis using the LASSO-Cox regression technique. The risk score was assessed based on the premise of directly combining the equation underneath with the mRNA expression level duplicated the multivariate Cox relapse coefficient (β) demonstrate, as we previously reported [14]. Risk score = ∑iCoefficient (mRNAi)*Expression (mRNAi). We stratified HCC patients into two subgroups due to the ideal hazard score edge. The prescient control and autonomy of the prognostic signature were evaluated by ROC examination, Kaplan–Meier survival examination and cox relative risks relapse investigation.

### Genetic alterations and functional analysis

The mutation data of HCC patients were downloaded to analyze the difference in genetic alterations between the different subgroups with R package “maftools”. Gene Set Enrichment Analysis (GSEA) was performed in the Metascape database to explore significantly altered GO and KEGG items [15, 16]. We have received permission from Kanehisa Laboratories.

### Immune cell infiltration and immune checkpoint gene analysis

Stromal and immune scores in tumor tissue were estimated by ESTIMATE based on gene expression profiles of HCC samples to assess the abundance of stromal and immune cells within the tumor [17]. Furthermore, to assess the distribution differences of tumor-infiltrating immune cells (TIICs) in the HCC TME in more detail, CIBERSORT [18], xCELL [19], MCPcounter [20], and TIMER [21] databases were used to measure the abundance ratio of TIICs. The tumor immune dysfunction and exclusion (TIDE) score was used to predict the immune checkpoint blockade (ICB) response in HCC patients [22]. Finally, we compared the expression differences of various immune checkpoint genes including PD1, PD-L1, CD276, CTLA4, LAG3, CXCR4, IL1A, IL6, TGFB1, TNFRSF4, TNFRSF9, and PD-L2 in different subgroups.

### Drug susceptibility analysis

The association between anticancer drug sensitivity and mRNA molecules in our risk model was directly explored in the CellMiner database [23]. 574 in advanced clinical trials and 216 Food and Drug Administration (FDA)-approved drugs were used for follow-up analyses. Drugs with adjusted *P*-value < 0.001 and Pearson correlation coefficient > 0.3 as cut-off criteria were considered tumor-sensitive drugs.

### Hypoxia model construction and gene expression validation by qRT-PCR

The recommended DMEM medium (Sangon Biotech, China) containing 10% fetal bovine serum (FBS, Sangon Biotech, Shanghai, China) was cultured in Hep3B and Huh7 cells obtained from the Cell Bank of Shanghai Institute of Cell Research, Chinese Academy of Sciences (Shanghai, China) at 100% humidity, 37 °C, and 5% CO2. Following a 24-h incubation period with two different oxygen concentrations (1 percent and 21 percent), the cells were removed, lysed, and the total RNA was extracted. To identify the variations in gene expression in the two HCC cells under different oxygen concentration culture conditions, the qRT-PCR (Sangon Biotech, China) approach was lastly employed. Primer sequences are shown in Table S2.

### Gene expression validation in clinical samples

To further validate the value of signature in predicting the prognosis of HCC, we collected tissues from 50 normal tissues and 59 HCC samples and used qRT-PCR (Sangon Biotech, China) to detect the expression of genes in signature [24], as previously done.

### Statistical analysis

**Fig. 1:**
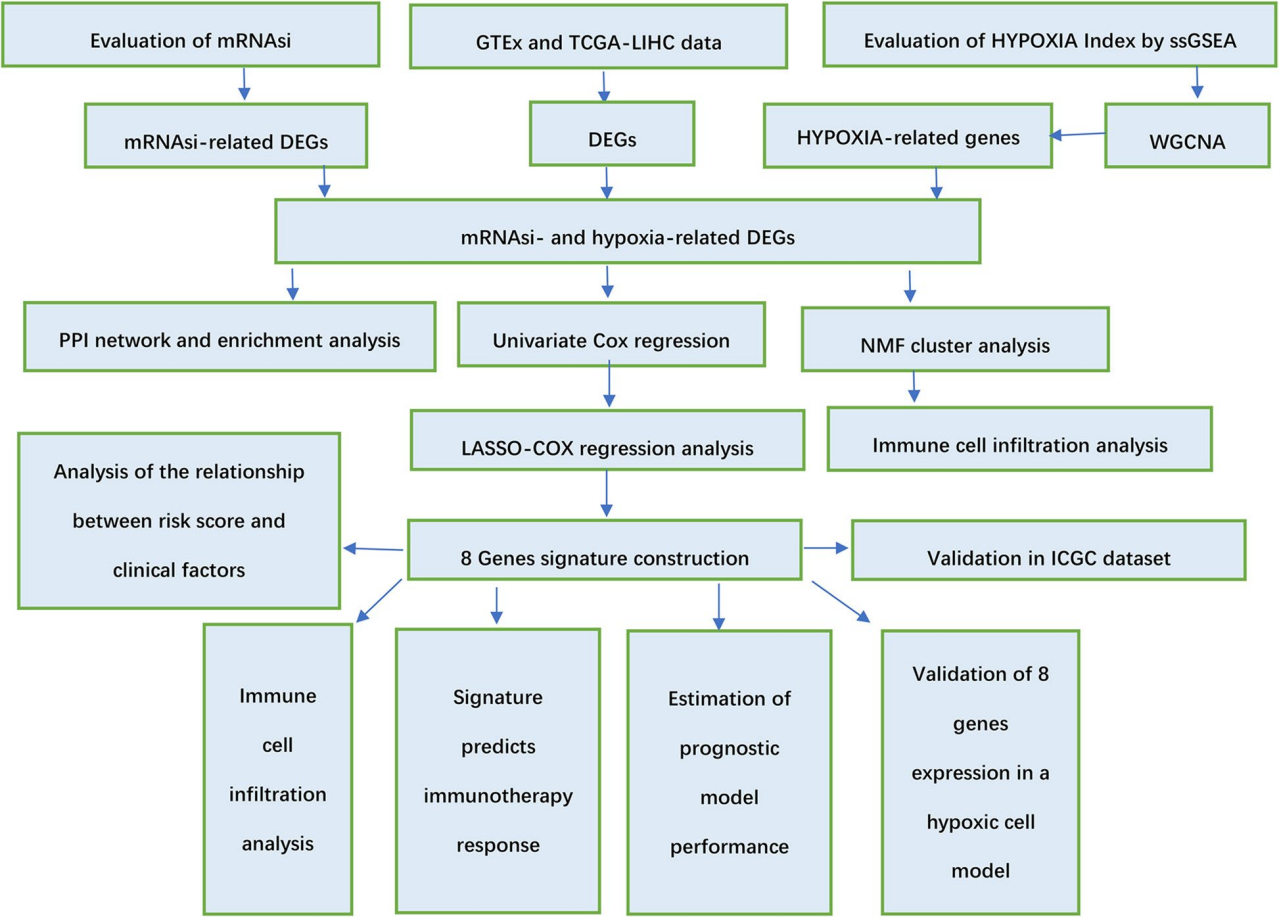
The flow chart

The grouped t-test or Mann-Withney-Wilcoxon test was used to evaluate continuously distributed numerical data, while the qualitative data were analyzed using the chi-square test. NMF cluster analysis was used to divide HCC patients into two groups with significant survival differences based on stemness-hypoxia-related DEGs by the NMF package. Kaplan–Meier survival analysis was used to estimate overall survival rates between groups using the survival and survminer packages. Receiver operating characteristic (ROC) curves were performed to calculate the AUC values at 1-, 2-, and 3-year using the timeROC package. Univariate and multivariate Cox regression analyses were performed to evaluate whether the signature was an independent prognostic factor, and a nomogram was constructed using the RMS package based on its results. Correlation analysis was performed using the Pearson or Spearman correlation test. When the P value was less than 0.05, the results were deemed statistically significant. All analyses were performed using the R programming language (version 4.1.2). The flowchart of this study is shown in Fig. 1.

## Results

### High mRNAsi in tumor samples predicts poorer patient survival

**Fig. 2:**
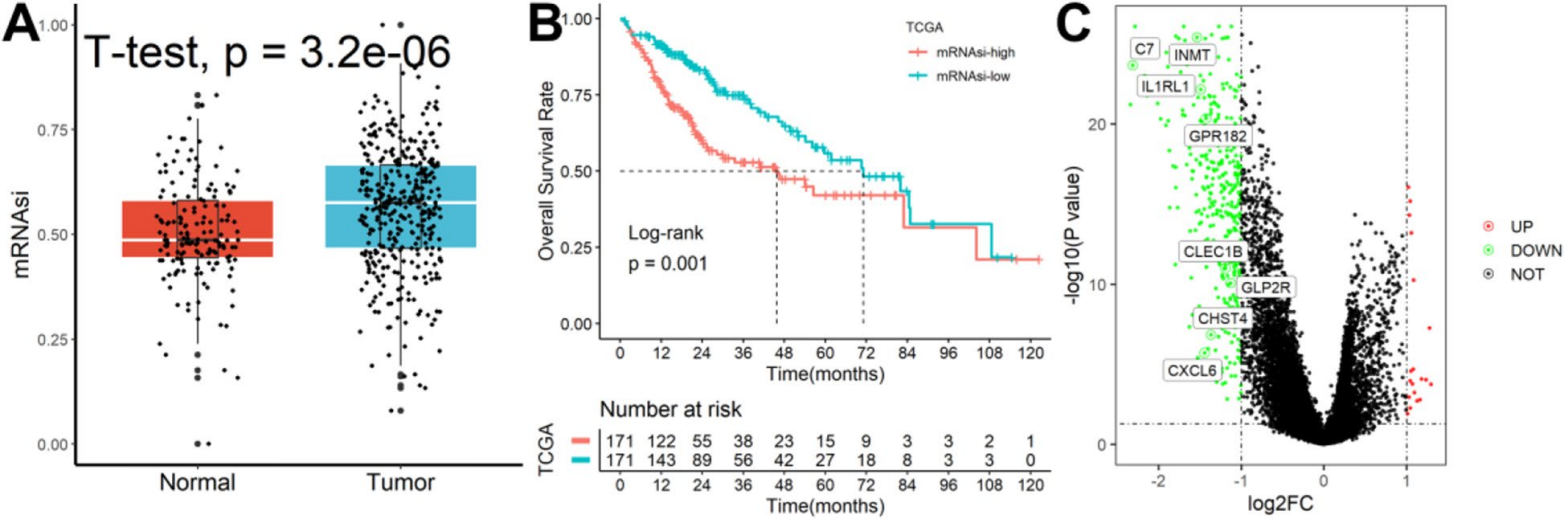
Identification of mRNAsi-related DEGs. **A** Normal samples had significantly lower mRNAsi values than HCC samples. **B** Significant survival differences between patients in high- and low-mRNAsi subgroups were observed. **C** 387 mRNAsi-related DEGs between high- and low-mRNAsi subgroups were identified

As shown in Fig. 2A, we found that normal samples had significantly lower mRNAsi values than HCC samples. Furthermore, significant survival differences between HCC patients in high- and low-mRNAsi subgroups were observed (Fig. 2B). Finally, we identified 387 mRNAsi-related DEGs between high- and low-mRNAsi subgroups (Fig. 2C).

### Identification of hypoxia-related genes

**Fig. 3:**
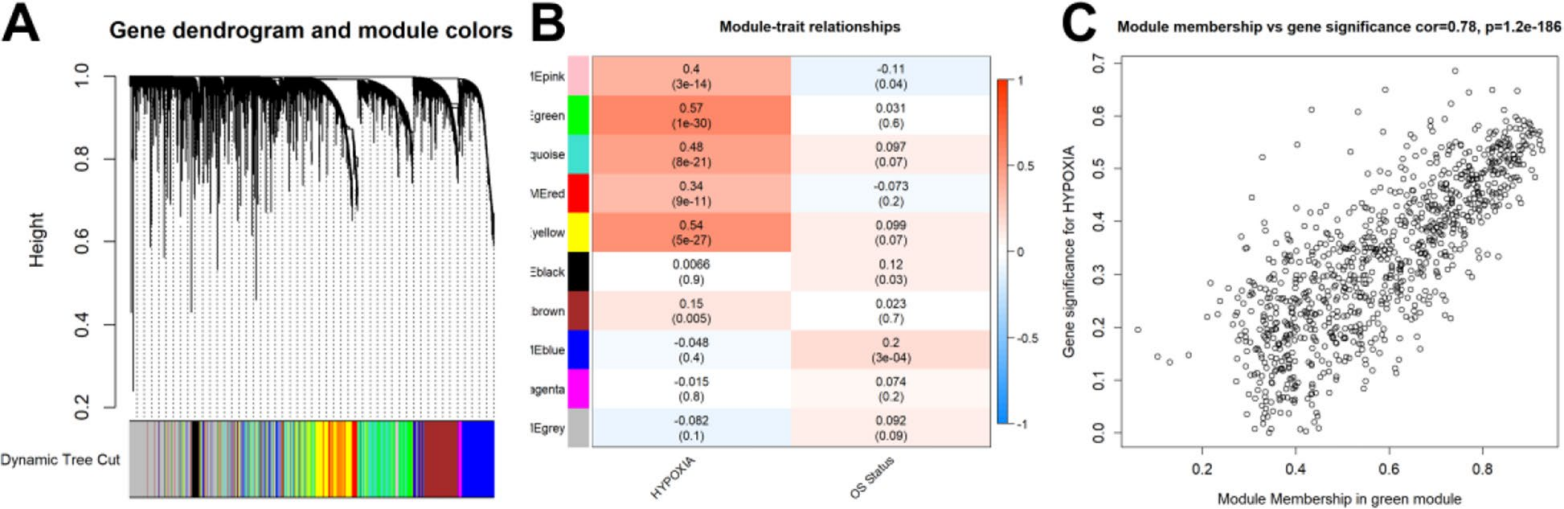
Identification of hypoxia-related genes by the WGCNA. **A** Nine non-grey modules were produced. **B**-**C** The green module had the strongest correlation with the hypoxic signature score

According to the outcomes of the WGCNA (Fig. 3A), nine non-grey modules were produced after the ssGSEA method assessed the hypoxia signature score and the removal of outliers (Supplementary Figure S1). The green module had the strongest correlation with the hypoxic signature score, as seen in Fig. 3B&C (R^2^ = 0.78, *P* = 1.2e-186), which contained 908 hypoxia-related genes.

### Molecular subtypes identification

**Fig. 4:**
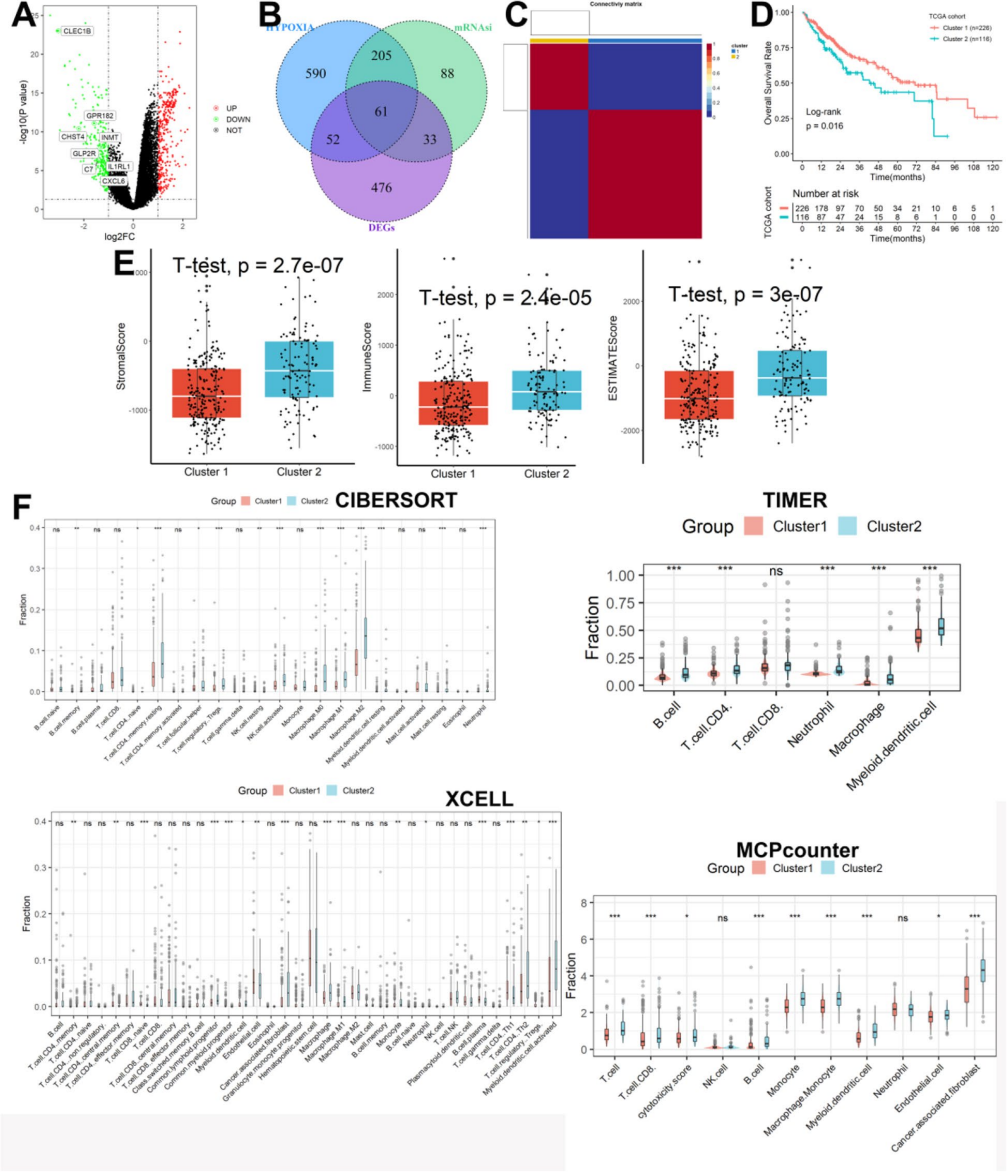
Molecular subtypes identification by the NMF algorithm. **A** 622 DEGs from the TCGA and GTEx datasets. **B** 61 overlapping genes were identified as SHRGs. **C** The optimal number of clusters was identified as two. **D** Significant survival differences between patients in the Cluster 1 and Cluster 2 subgroups were observed. **E** Samples from Cluster 1 had lower immune, stromal, and ESTIMATE scores compared with samples from Cluster 2. **F** Analysis of immune infiltrating cells

After obtaining 622 DEGs from the TCGA and GTEx datasets (Fig. 4A), 61 overlapping genes were identified as SHRGs (Fig. 4B) and were used for subsequent analysis. The expression levels of 61 SHRGs in normal and tumor tissues were shown in Supplementary Figure S2A. We also constructed a protein–protein interaction (PPI) network based on these genes (Supplementary Figure S2B). Furthermore, the main enriched entries for these genes were NABA matrisome associated, blood vessel development, in-utero embryonic development, and malignant pleura mesothelioma (Supplementary Figure S2C). The optimal number of clusters was identified as two based on cophenetic, dispersion, and profile (Fig. 4C, Supplementary Figure S3). Significant survival differences between patients in the Cluster 1 and Cluster 2 subgroups were observed (Fig. 4D). As shown in Fig. 4E, samples from Cluster 1 had lower immune, stromal, and ESTIMATE scores compared with samples from Cluster 2. In addition, as shown in Fig. 4F, according to the CIBERSORT, patients in the Cluster 2 had higher abundance levels of memory B cells, resting memory CD4 T cells, follicular helper T cells, Tregs, active NK cells, M0 macrophage, M1 macrophage, M2 macrophage, resting myeloid dendritic cells, resting mast cells, and neutrophil, and lower abundance levels of naive CD4 T cells when compared with patients in the Cluster 1. According to the TIMER database, patients in the Cluster 2 had higher abundance levels of B cells, CD4 T cells, neutrophil cells, macrophage, and myeloid dendritic cells when compared with patients in Cluster 1. According to the xCELL database, patients in the Cluster 2 had higher abundance levels of memory CD4 T cells, naive CD8 T cells, common lymphoid progenitor, myeloid dendritic cell, cancer-associated fibroblast, macrophage, macrophage M1, neutrophil, Th2 CD 4 T cell, and activated myeloid dendritic cell, and lower abundance levels of central memory CD4 T cells, common myeloid progenitor, endothelial cell, plasma B cell, and Th1 CD 4 T cell when compared with patients in the Cluster 1. According to the MPCcounter database, patients in the Cluster 2 had higher abundance levels of T cells, CD8 T cells, B cells, cancer-associated fibroblast, monocyte, macrophage monocyte, myeloid dendritic cell, neutrophil, and endothelial cell when compared with patients in the Cluster 1. When considering the four algorithms comprehensively, as shown in Table S3, myeloid dendritic cells, macrophages, neutrophils, and CD4 T cells were differential immune cells between Cluster 1 and Cluster 2 groups based on four algorithms. Finally, we found the mutation rates of the top fifteen most significantly mutated genes were significantly different in the two subgroups (Supplementary Figure S4).

### Establishment of a signature for tumor stemness and hypoxia characteristics in HCC

**Fig. 5:**
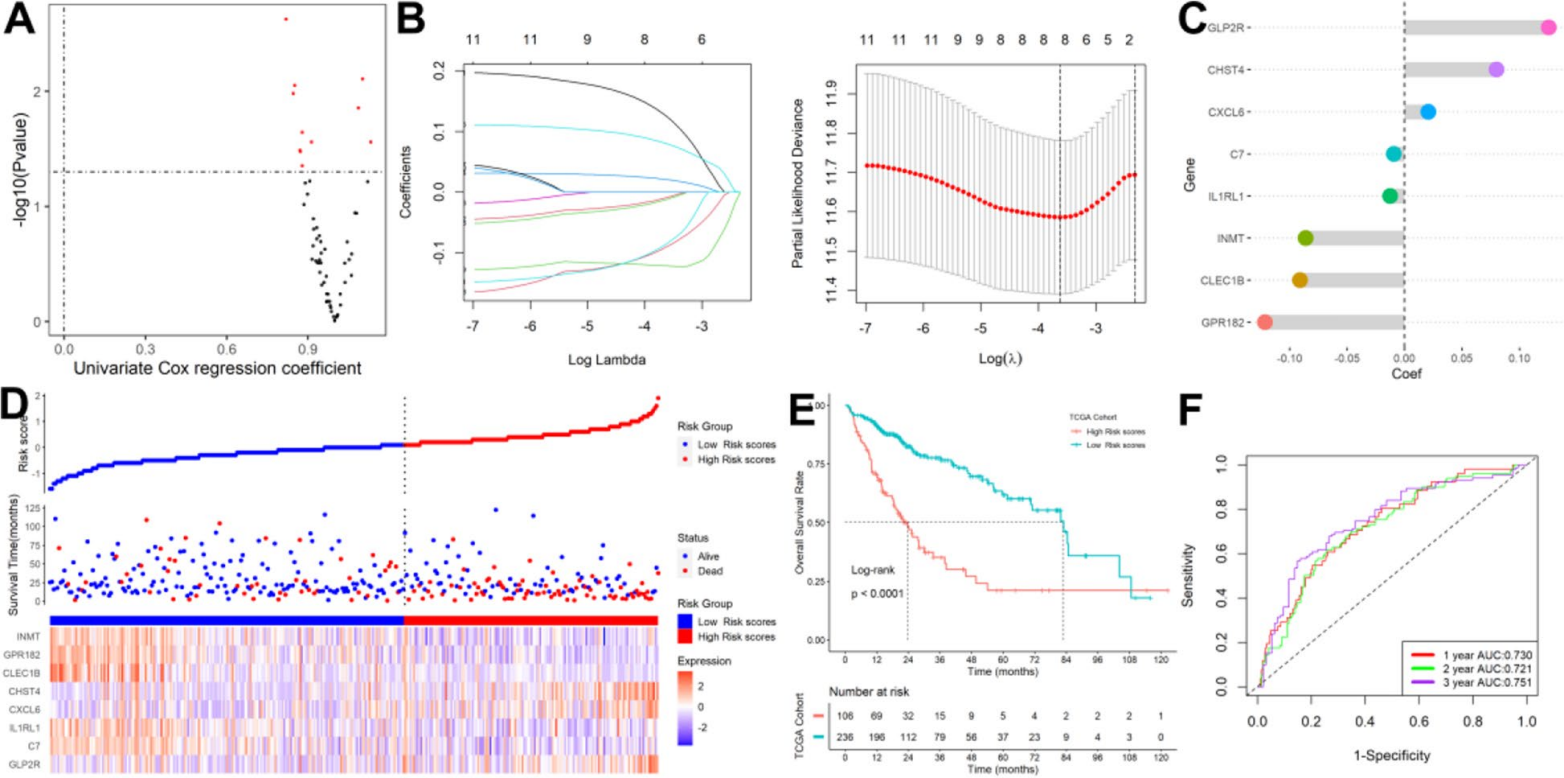
Establishment of a signature for tumor stemness and hypoxia characteristics. **A** Univariate Cox regression analysis. **B** Eleven prognostic SHRGs were selected by the LASSO-Cox regression. **C** The coefficients for each gene in the multivariate Cox proportional model. **D** Patients were stratified into high- or low-risk subgroups with an optimal risk score threshold. **E** Patients with higher risk scores were significantly relevant to poorer survival outcomes. **F** ROC analysis

**Fig. 6:**
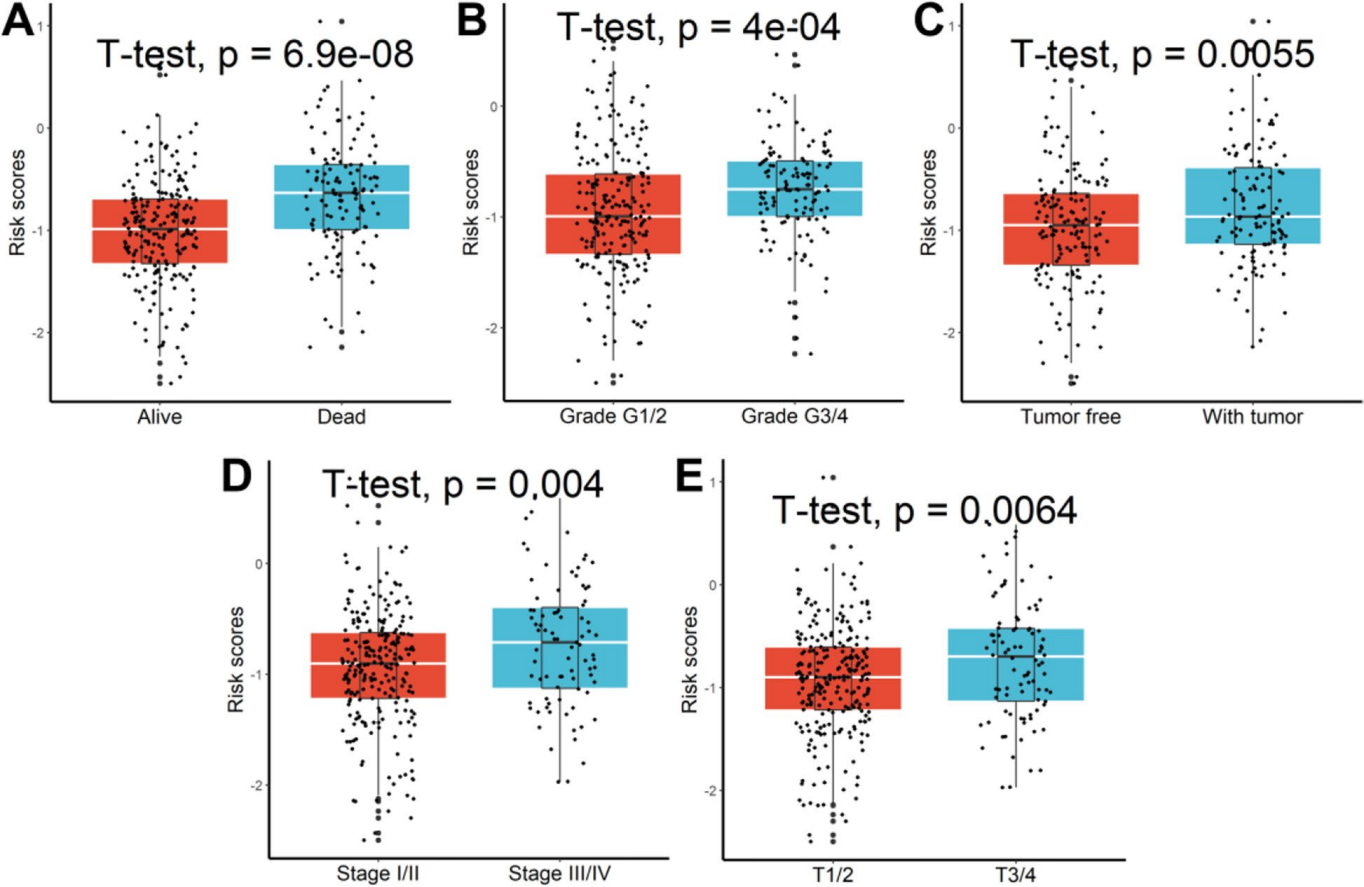
Higher risk scores were linked to patient death **A**, later grade **B**, recurrence **C**, advanced TNM stage **D**, and later T stage **E**

The prognostic value of the 61 SHRGs was calculated in a univariate Cox regression model (Fig. 5A). Eight of the eleven prognostic SHRGs were screened out by performing the LASSO-Cox regression model (Fig. 5B) and were then put into a multivariate Cox proportional model (Fig. 5C). Risk score = (0.19418143 × GLP2R)—(0.03421663 × C7) – (0.04084137 × IL1RL1) + (0.03137575 × CXCL6) + (0.11103071 × CHST4)—(0.14224683 × CLEC1B)—(0.11412072 × GPR182)—(0.14543623 × INMT). The aforementioned algorithm was used to construct risk scores for HCC patients, and an ideal risk score threshold was used to categorize patients into high- or low-risk categories (Fig. 5D). Patients with greater risk ratings had considerably worse survival outcomes (Fig. 5E). This classifier demonstrated strong prognostic performance with AUCs at 1-, 2-, and 3-year of 0.730, 0.721, and 0.751. (Fig. 5F). Higher risk scores were further associated with patient mortality (Fig. 6A), later grade (Fig. 6B), relapse (Fig. 6C), progressed TNM stage (Fig. 6D), and later T stage (Fig. 6E). The results demonstrated that this predictive model could further distinguish individuals with various clinical features when additional stratified survival analysis was done for those clinical variables (Supplementary Figure S5). Last but not least, univariable Cox regression analysis showed that this classifier was statistically related to HCC patient survival outcomes (HR = 4.103, 95%CI 2.648–6.359, *P* = 2.6e-10). After controlling for other clinical parameters, multivariate Cox regression analysis showed that the statistically significant variables collected above might be used as an independent prognostic factor for HCC patients (HR = 3.318, 95%CI 2.114–5.214, *P* = 1.9e-07).

### Functional and genetic alterations analysis

Gene set enrichment analysis (GSEA) between high- and low-risk score subgroups was used to identify GO and KEGG items with FDR less than 0.05, which were mostly engaged in cell cycle and metabolic processes (Supplementary Figure S6). The top fifteen substantially modified genes were then subjected to a genetic alteration study, which revealed that the mutation rates in the two groupings were considerably different. The most often altered gene in the high-risk score subgroup was TP53 (43%) and the most altered gene in the low-risk score subgroup was CTNNB1 (230%). (Supplementary Figure S7).

### Verification of the signature in the ICGC cohort

**Fig. 7:**
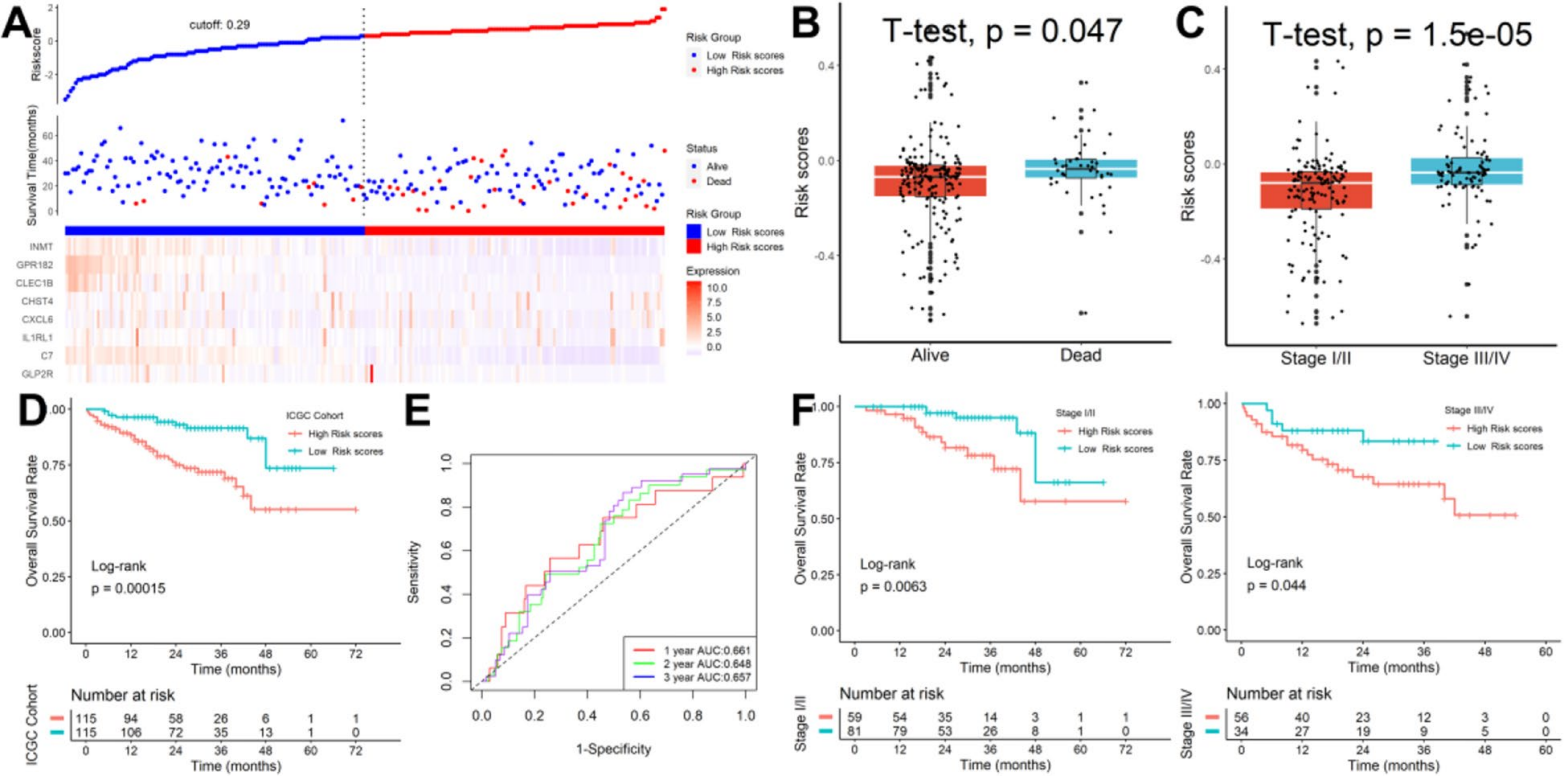
Verification of the signature in the ICGC cohort. **A** Patients were stratified into high- or low-risk subgroups. Patients who are dead (**B**) or with advanced TNM stages (**C**) had significantly higher scores. **D** Kaplan–Meier survival analysis revealed that patients with higher risk scores were prominently relevant to poorer OS rates. **E** ROC analysis. **F** This prognostic model could further differentiate patients of different ages and TNM stages

Risk scores of patients were calculated with the same formula, and patients were stratified into high- or low-risk subgroups in the ICGC cohort (Fig. 7A). Patients who are dead (Fig. 7B) or with advanced TNM stages (Fig. 7C) had significantly higher scores. Kaplan–Meier survival analysis revealed that patients with higher risk scores were prominently relevant to poorer OS rates (Fig. 7D) and ROC analysis revealed that this signature had a good prognostic performance with AUCs at 1-, 2-, 3-year of 0.661, 0.648, 0.657 (Fig. 7E). In addition, this prognostic model could further differentiate patients of different TNM stages (Fig. 7F).

### Analysis of immune infiltrating cells

**Fig. 8:**
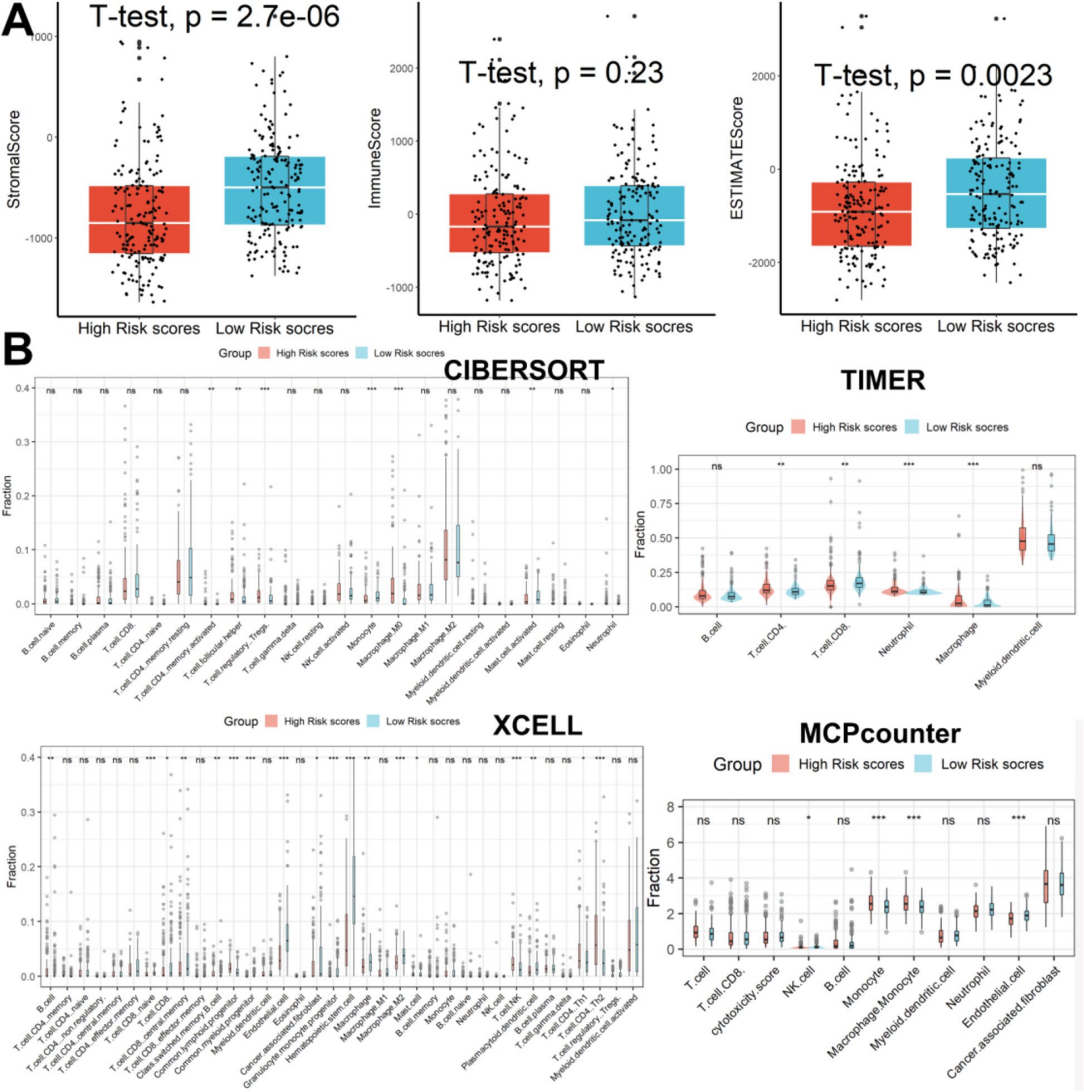
Analysis of immune infiltrating cells. **A** Samples in high-risk groups had lower stromal, and ESTIMATE scores compared with samples in low-risk groups, however, there was no difference in immune scores between the two groups. **B** Differential analysis of immune infiltrating cells

As shown in Fig. 8A, samples in high-risk groups had lower stromal, and ESTIMATE scores compared with samples in low-risk groups, however, there was no difference in immune scores between the two groups. In addition, as shown in Fig. 8B, according to the CIBERSORT, patients in the high-risk score group had higher abundance levels of activated memory CD4 T cells, follicular helper T cells, Tregs, M0 macrophage, and neutrophil, and lower abundance levels of monocyte and activated mast cells when compared with patients in the low-risk score group. According to the TIMER database, patients in the high-risk score group had higher abundance levels of CD4 T cells, macrophages, and neutrophils, and lower abundance levels of CD8 T cells when compared with patients in the low-risk score group. According to the xCELL database, patients in the high-risk score group had higher abundance levels of class-switched memory B cell, common lymphoid progenitor, common myeloid progenitor, mast cell, NK T cell, Th1 CD4 T cell, and Th2 CD4 T cell, and lower abundance levels of naïve CD8 T cell, CD8 T cell, central memory CD8 T cell, endothelial cell, cancer-associated fibroblast, granulocyte monocyte progenitor, hematopoietic stem cell, macrophage, macrophage M2, and plasmacytoid dendritic cell when compared with patients in the low-risk score group. According to the MPCcounter database, patients in the high-risk score group had higher abundance levels of monocyte and macrophage monocyte, and lower abundance levels of NK cell and endothelial cells when compared with patients in the low-risk score group. When considering the four algorithms comprehensively, as shown in Table S4, macrophage was the differential immune cell between high- and low-risk score groups based on four algorithms.

### Establishment of a nomogram model and drug susceptibility analysis

A nomogram model was built in the TCGA dataset to investigate the effectiveness of this classifier's coefficient prediction. The results showed that the nomogram with a C-index of 0.803 could aid in the development of an accurate quantitative method for predicting the 1-, 2-, and 3-year survival rates (Supplementary Figure S8A). The calibration curves' overlap between the predicted and actual probability of 1-, 2-, and 3-year survival rates showed good agreement (Supplementary Figure S8B). Among the 574 in advanced clinical trials and 216 FDA-approved drugs, 77 were considered tumor-sensitive drugs (Table S5), and the top 16 most significant tumor-sensitive drugs were shown in Supplementary Figure S9.

### Forecasting ICB response by the 8-gene signature

**Fig. 9:**
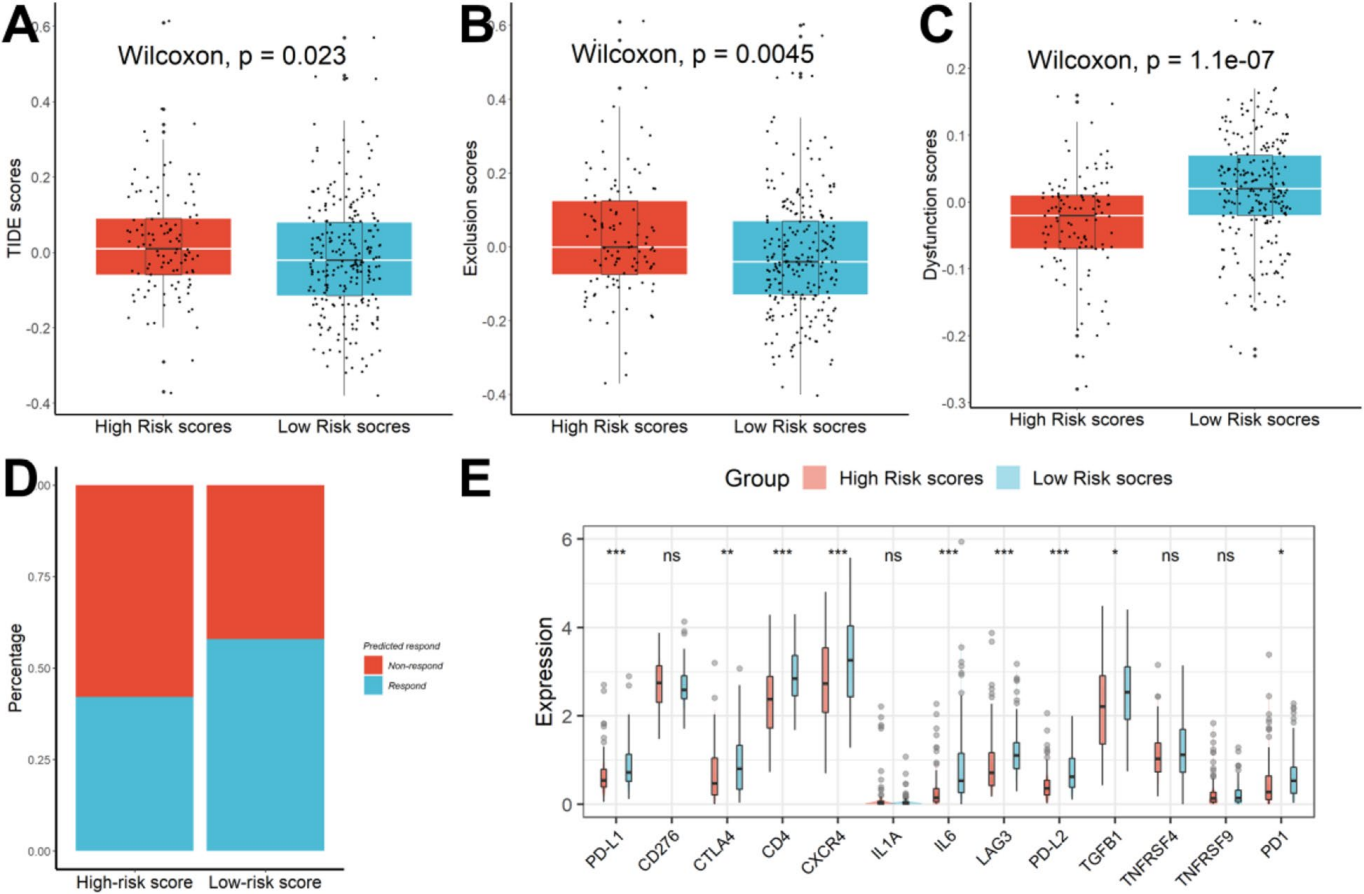
Forecasting ICB response. **A** The TIDE scores in the high-risk scores group were much higher than that in the low-risk scores group. **B** T-cell exclusion scores were lower in the high-risk scores group than that in the low-risk scores group. **C** T-cell dysfunction scores were greater in the high-risk scores group than that in the low-risk scores group. **D** The proportion of “respond” in the high-risk group was lower than that in the low-risk score group. **E** Patients in the high-risk scores group had higher expression of CD276, CTLA4, IL1A, TGFB1, TNFRSF4, and TNFRSF9

We normalized the TCGA transcriptome data of 342 HCC patients, that is, the expression of each gene minus the average value of the gene in all samples, and then imported the data into the TIDE (http://tide.dfci.harvard. edu/) database. The analysis results were finally downloaded. We found that the TIDE scores in the group with higher risk scores were significantly greater than those in the group with lower risk scores, as shown in Fig. 9A. The high-risk scores group had higher T-cell exclusion values than the low-risk scores group (Fig. 9B), while the low-risk scores group had higher T-cell dysfunction scores than the high-risk scores group (Fig. 9C). When the predicted response rate to immunotherapy was taken into account, the proportion of “respond” in the high-risk group was even lower (Fig. 9D). Additionally, we discovered that patients with lower risk ratings had greater levels of the genes PD-L1, CTLA4, CD4, CXCR4, IL6, LAG3, TGFB1, PD1, and PD-L2 than patients with higher risk scores in the ICGC dataset (Fig. 9E), indicating that these ICIs may be more beneficial for patients with lower risk scores.

### Expression levels of these genes in hypoxia cell models

**Fig. 10:**
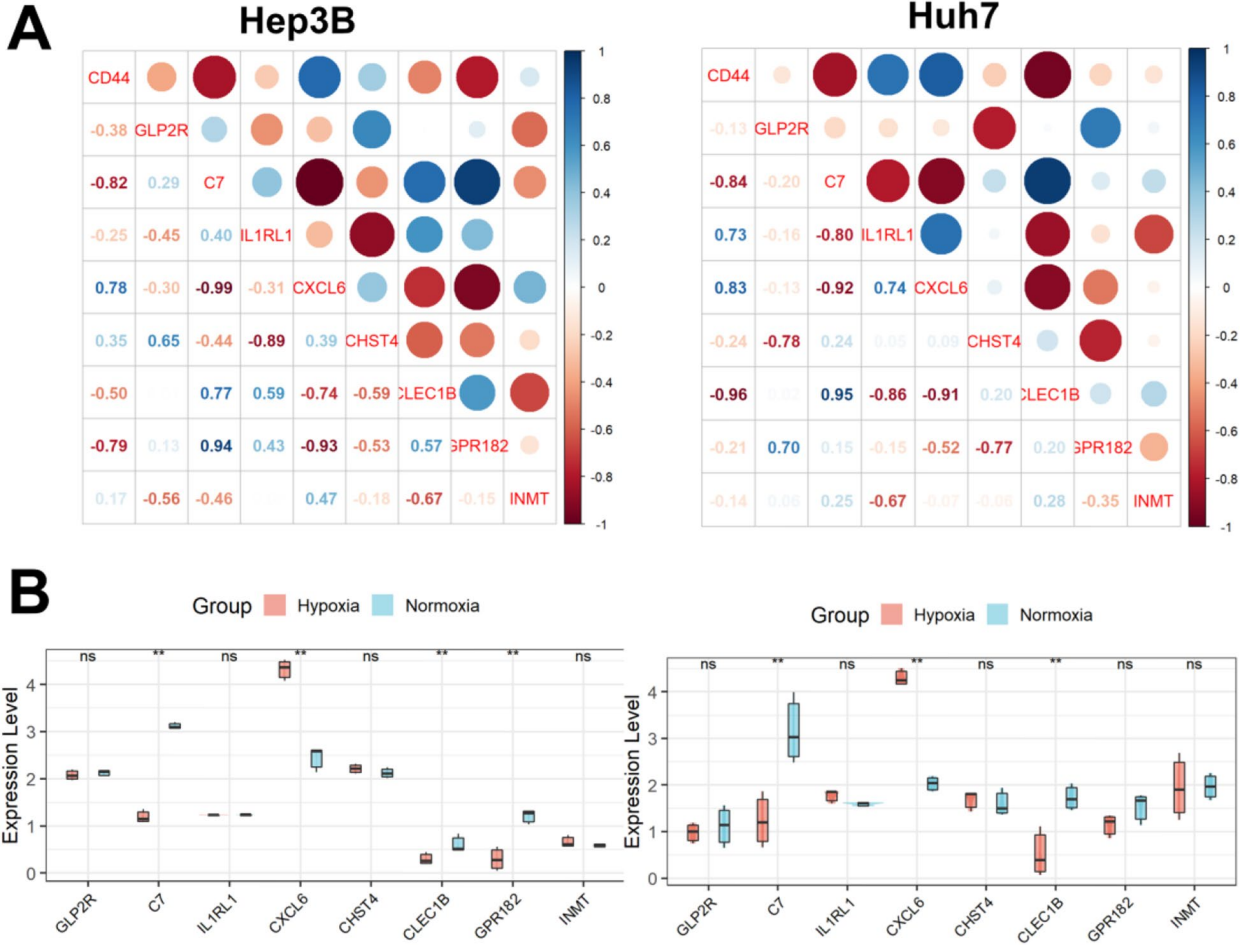
Expression levels of these genes in hypoxia cell models. **A** CD44 was significantly associated with C7, CXCL6, CLEC1B, and GPR182 in Hep3B cells and with C7, CXCL6, CLEC1B, and IL1RL1 in Huh7 cells. **B** Compared with the culture condition with 21% oxygenation concentration, after 24 h of culture at 1% oxygenation concentration, the expression of C7, CLEC1B, and GPR182 in Hep3B cells was significantly decreased, and the expression of CXCL6 was significantly increased in Hep3B cells, while in Huh7 cells, the expressions of C7 and CLEC1B were significantly decreased, and the expression of CXCL6 was significantly increased

As shown in Fig. 10A, CD44, a putative tumor stem cell marker, was significantly associated with C7, CXCL6, CLEC1B, and GPR182 in Hep3B cells and with C7, CXCL6, CLEC1B, and IL1RL1 in Huh7 cells. Compared with the culture condition with 21% oxygenation concentration, after 24 h of culture at 1% oxygenation concentration, the expression of C7, CLEC1B, and GPR182 in Hep3B cells was significantly decreased, and the expression of CXCL6 was significantly increased in Hep3B cells, while in Huh7 cells, the expressions of C7 and CLEC1B were significantly decreased, and the expression of CXCL6 was significantly increased (Fig. 10B). All of the above indicated that C7, CLEC1B, and CXCL6 were not only related to the tumor stemness but also related to hypoxia.

### Expression and prognostic value of C7, CLEC1B, and CXCL6

**Fig. 11:**
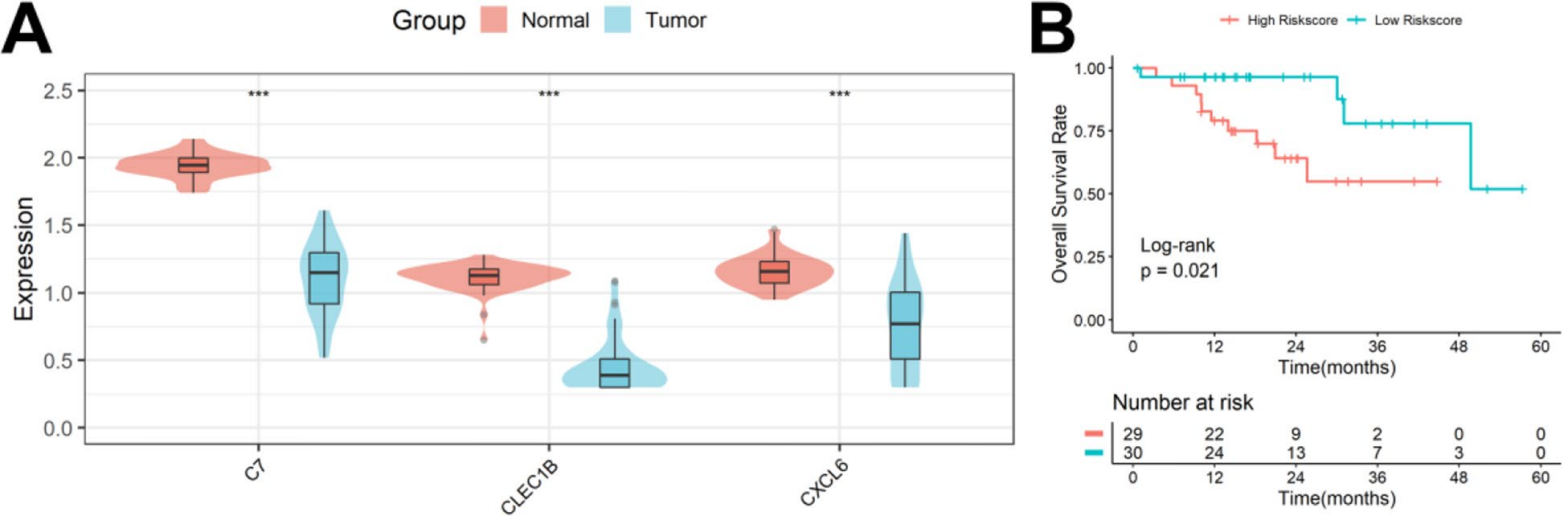
Expression and prognostic value of C7, CLEC1B, and CXCL6. **A** All three genes were down-regulated in HCC tissues compared to normal tissues. **B** The survival rate of patients with higher risk scores was poorer than that of patients with lower risk scores

Based on the above cell culture results, only C7, CLEC1B, and CXCL6 were associated not only with tumor stemness but also with hypoxia; therefore, we focused on exploring the expression levels and prognostic value of these three genes. As shown in Fig. 11A, all three genes were down-regulated in HCC tissues compared to normal tissues. After substituting the expression of the three genes into the formula to calculate the risk score, the survival rate of patients with higher risk scores was poorer than that of patients with lower risk scores (Fig. 11B).

## Discussion

Although heterogeneous CSCs with different differentiation statuses are not abundant in tumor tissues, their differentiation potential, unlimited proliferation, and self-renewal abilities are closely related to oncogenesis, tumor advancement, relapse, and drug resistance [25]. Accumulating evidence suggests that multiple signaling pathways, including Wnt/-catenin, Notch, Hypoxia, and STAT3, play critical roles in maintaining self-renewal in CSCs and participating in tumorigenesis [26]. Due to the uneven distribution of newly formed microvessels in tumor tissue, and the oxygen consumption of tumor cells being higher than that of normal cells, HCC tissue is often in a state of hypoxia [27]. Intratumoral hypoxia can in turn further promote tumor malignancy and aggressiveness [28, 29]. However, few studies have combined tumor stemness and hypoxia phenotypes to construct a model and explore mRNAsi and hypoxia-related genes in HCC. Therefore, an in-depth analysis of the relationship between HCC stemness and hypoxia and the effectiveness of immunotherapy, and the exploration of methods for subtype differentiation based on tumor stemness- and hypoxia-related genes, are necessary to explore potential therapeutic targets to improve the poor prognosis of HCC patients.

In this study, after identifying tumor stemness- and hypoxia-related genes through a series of bioinformatics analyses, we constructed a prognostic stratification model based on these SHRGs, which can be effectively applied to the prognostic classification of HCC patients and the prediction of ICB efficacy. Independent validation was performed using patient samples from the ICGC cohort and a clinical sample cohort, and the validation results were favorable. In addition, we also constructed hypoxic cell models in Herp3B and Huh7 cells to verify the expression of genes in the prognostic model and found that C7, CLEC1B, and CXCL6 were not only related to the tumor stemness but also related to hypoxia, suggesting that these three genes can serve as therapeutic targets for tumor stemness and hypoxia targeting.

CXCL6 is involved in the development of various diseases including tumors [30–33]. CXCL6 secreted by HCC cells can increase the HCC stemness and accelerate the progression of HCC by activating the extracellular signal-regulated kinase (ERK) 1/2 signaling pathway of tumor-associated fibroblasts (CAFs) [34, 35]. Hypoxia induces increased expression of multiple HIF-1-dependent CXC chemokines, including CXCL6, in HCC cells [36]. These suggest that CXCL6 may promote the progression of HCC by simultaneously affecting the stemness and oxygenation status of tumor cells. C7 is a complement protein that is differentially expressed in a variety of tumors and is associated with poor patient prognosis [37–39]. C7 can act as an anti-cancer gene to inhibit HCC metastasis by targeting the HGF signaling pathway [40]. Paradoxically, Hyang found that C7 could control tumor cell stemness by regulating the expression of LSF-1, and overexpressed C7 can increase stemness factor secretion and promote tumor cell proliferation [41], which suggests that C7 may be a tumor-promoting gene. Hypoxia inhibits innate immune processes in Larimichthys crocea and reduces most complement components including C7 [42], but whether hypoxia affects the expression of C7 in humans has not yet been reported. CLEC1B is an HCC prognostic marker worthy of further exploration for its great potential [43]. Low expression of CLEC1B can significantly promote the growth rate of HCC cells, shorten the tumor doubling time, and aggravate the poor prognosis of patients [44]. Low expression of CLEC1B can also predict the clinical outcome of HCC patients with tumor hemorrhage [45]. In addition, CLEC1B was also associated with inflammatory immune cell infiltration within the HCC TME [46]. In our present study, CLEC1B was significantly negatively correlated with the expression of tumor stemness marker CD44, and its expression was significantly reduced under hypoxic culture conditions in Hep3B and Huh7 cells.

Growing evidence suggests that the TME plays an unparalleled role in the initiation and progression of HCC, as do hypoxia and tumor stemness. A variety of cell types, including cancer cells, immune cells, stromal cells, endothelial cells, complex cytokine secretion, and fibroblasts, are present within TME and can be involved in tumor formation, progression, and migration [47]. Macrophages with intra-hypoxic microenvironment targeting are widely distributed in high-risk score samples and play an important role in hypoxia-mediated immune evasion. When the results of the four algorithms TIMER, CIBERSORT, XCELL, and MCPcounter were considered together, macrophages were the differential immune cells between the high- and low-risk subgroups, suggesting that the stemness-hypoxia-related signature may promote poor prognosis of HCC by modulating macrophage infiltration. Meanwhile, regulatory T cells and neutrophils in high-scoring samples can form extracellular traps to jointly promote the transformation of non-alcoholic steatohepatitis to HCC [48]. In addition, we also observed a higher mutation rate of the TP53 gene in samples with high-risk scores, which is consistent with previous reports that TP53 mutation is a genomic consequence of tumor hypoxia [49]. Complex mechanisms of interaction between immune cells and effector molecules in TME can promote or inhibit the growth of HCC by altering the immune system. A variety of approved or pending systemic therapies targeting typical tumor-associated pathways in TME, including vascular endothelial growth factor (VEGF)-dependent angiogenesis, adenosine 5′ monophosphate-activated protein kinase (AMPK), and PI3K/AKT/rapamycin (mTOR) mammalian targets, have a limited role in HCC [50]. Given the potent cytotoxicity of T lymphocytes in cancer, TME-targeted therapies based on innate T cell immune responses, including ICB and chimeric antigen receptor (CAR) T cell therapies, are in full swing in current research [51]. CAR-T therapy can identify tumor-associated antigen (TAA) and effectively eliminate tumor cells through a non-MHC approach, showing excellent therapeutic effects in hematological malignancies [52]. Excitingly, glypican-3 (GPC-3) can act as an anchoring carcinoembryonic proteoglycan on tumor cell membranes in combination with CAR-T technology to provide a new light for immunotherapy of solid tumors, especially HCC [53]. GPC3-CAR-T cells have been shown to effectively inhibit tumor growth in HCC xenograft models [54]. In addition, a variety of immune checkpoint inhibitors (ICIs), including PD-1 and CTLA-4, are highly anticipated in the treatment of HCC [55]. In our study, we discovered that patients with lower risk ratings had greater levels of the genes PD-L1, CTLA4, and PD1 than patients with higher risk scores, indicating that these ICIs may be more beneficial for patients with higher risk scores. It is undeniable that although ICIs can suppress the immune system by blocking the expression of CTLA-4 or PD-1 and thus produce a very durable antitumor response, the immune-related adverse events (IRAEs) generated can interfere with the effectiveness of immunosuppressive therapy [56]. Therefore, ICIs combined with GPC3-CAR-T therapy may bring unexpected results to HCC patients.

The TIDE algorithm could find biomarkers to predict the efficacy of ICBs through a comprehensive analysis of hundreds of different tumor expression profiles, and its prediction effect is significantly better than the existing biomarkers. Although the developers of the TIDE algorithm state that it may not apply to other therapies than melanoma and non-small cell lung cancer (NSCLC), many studies have shown that the application of the TIDE algorithm can be extended to other tumors besides melanoma and NSCLC, such as HCC [57], breast cancer [58], head and neck squamous cell carcinoma [59], and other tumors [60]. The TIDE algorithm can well help us estimate the immunotherapy response of HCC patients [61, 62]. In this study, we found that the TIDE scores in the group with higher risk scores were significantly greater than those in the group with lower risk scores, and the proportion of “respond” in the high-risk group was even lower when the predicted response rate to immunotherapy was taken into account. In addition, considering that CSCs were associated with tumor resistance, and hypoxia promoted CSCs metastasis and aggravates drug resistance, we compared the expression levels of various ICBs between the two groups and found that patients with lower risk ratings had greater levels of the genes PD-L1, CTLA4, CD4, CXCR4, IL6, LAG3, TGFB1, PD1, and PD-L2 than patients with higher risk scores. All these indicated that these ICBs may be more beneficial for patients with lower risk scores and this signature can effectively predict immunotherapy response.

Although there have been several similar papers using stemness-hypoxia-related features to predict patient prognosis and discuss associations with immunotherapy, our manuscript still has some highlights. First, for the first time in HCC, we combined two phenotypes, hypoxia and tumor stemness, for patient prognostic analysis. Secondly, we constructed the signature with a higher prognostic value. Third, we validated the relationship between the genes in the signature and hypoxia and tumor stemness by cytological experiments. Finally, our constructed signature was validated in a clinical sample cohort and the validation results performed well. Admittedly, our study has some limitations. We need large multicenter randomized controlled studies to evaluate this signature in the future. In addition, the expression and prognostic predictive role of these eight genes at the protein level and their specific mechanisms in HCC need to be further evaluated in the future by additional in vivo and in vitro experiments.

## Conclusion

In conclusion, we constructed a stemness-hypoxia-related prognostic signature that can be used to predict the efficacy of ICIs therapy. We also confirmed that C7, CLEC1B, and CXCL6 were indeed associated with stemness and hypoxia by constructing hypoxic cell models. These may provide new ideas for individualized treatment of immunotherapy.

## Supplementary Information

**Additional file 1:** **Figure S1.** Identify the weighted value β that meets the law of scale-free networks. **Figure S2.** (A) Heatmap of the expression levels of 61 SHRGs in normal and tumor tissues. (B) Protein-protein interaction (PPI) network construction. (C) The main enriched entries for these genes. **Figure S3.** NMF rank survey. **Figure S4.** The mutation rates of the top fifteen most significantly mutated genes were significantly different between the Cluster 1 and Cluster 2 subgroups. **Figure S5.** The prognostic model could further differentiate patients with different clinical characteristics. **Figure S6.** Identification of GO and KEGG enrichment between high- and low-risk scores subgroups. **Figure S7.** The mutation rates of the top fifteen most significantly mutated genes were significantly different between high- and low-risk scores subgroups. **Figure S8.** The predictive significance of the prognostic model was verified in the nomogram. **Figure S9.** Top 16 most important tumor-sensitive drugs. **Table S1.** Clinical characteristics of HCC patients involved in the study. **Table S2.** The sequences of the qRT-PCR primers used in this study. **Table S3.** Immune cells with differences in abundance between Cluster1 and Cluster 2 by the four algorithms. **Table S4.** Immune cells with differences in abundance between high- and low-risk score groups by the four algorithms. **Table S5.** 77 tumor-sensitive drugs targeting tumor cell stemness.

## Data Availability

The datasets used and/or analyzed during the current study are available from the corresponding author upon reasonable request.

## Abbreviations

CSCs: Cancer stem cells
HCC: Hepatocellular carcinoma
TME: Tumor microenvironment
HIF: Hypoxia-inducible factor
DEGs: Differentially expressed genes
OCLR: One-class logistic regression
WGCNA: Weighted gene co-expression network analysis
GS: Gene significance
NMF: Non-negative matrix factorization
SHRGs: Stemness- and hypoxia-related genes
GSEA: Gene set enrichment analysis
TIICs: Tumor-infiltrating immune cells
TIDE: Tumor immune dysfunction and exclusion
FDA: Food and drug administration
FBS: Fetal bovine serum
ROC: Receiver operating characteristic curve
PPI: Protein–protein interaction
ERK: Extracellular signal-regulated kinase
CAFs: Tumor-associated fibroblasts
LASSO: Least absolute shrinkage and seletion operator
NMF: Non-negative matrix decomposition
