# Supplementary material for: A novel stemness-hypoxia-related signature for prognostic stratification and immunotherapy response in hepatocellular carcinoma
Source: BMC Cancer. 2022 Oct 28;22:1103. doi: 10.1186/s12885-022-10195-1 (PMC9617384; doi:10.1186/s12885-022-10195-1)
Supplement: Supplementary file 1 — Additional file 1: Figure S1. Identify the weighted value β that meets the law of scale-free networks. Figure S2. (A) Heatmap of the expression levels of 61 SHRGs in normal and tumor tissues. (B) Protein-protein interaction (PPI) network construction. (C) The main enriched entries for these genes. Figure S3. NMF rank survey. Figure S4. The mutation rates of the top fifteen most significantly mutated genes were significantly different between the Cluster 1 and Cluster 2 subgroups. Figure S5. The prognostic model could further differentiate patients with different clinical characteristics. Figure S6. Identification of GO and KEGG enrichment between high- and low-risk scores subgroups. Figure S7. The mutation rates of the top fifteen most significantly mutated genes were significantly different between high- and low-risk scores subgroups. Figure S8. The predictive significance of the prognostic model was verified in the nomogram. Figure S9. Top 16 most important tumor-sensitive drugs. Table S1. Clinical characteristics of HCC patients involved in the study. Table S2. The sequences of the qRT-PCR primers used in this study. Table S3. Immune cells with differences in abundance between Cluster1 and Cluster 2 by the four algorithms. Table S4. Immune cells with differences in abundance between high- and low-risk score groups by the four algorithms. Table S5. 77 tumor-sensitive drugs targeting tumor cell stemness. [file 12885_2022_10195_MOESM1_ESM.docx]

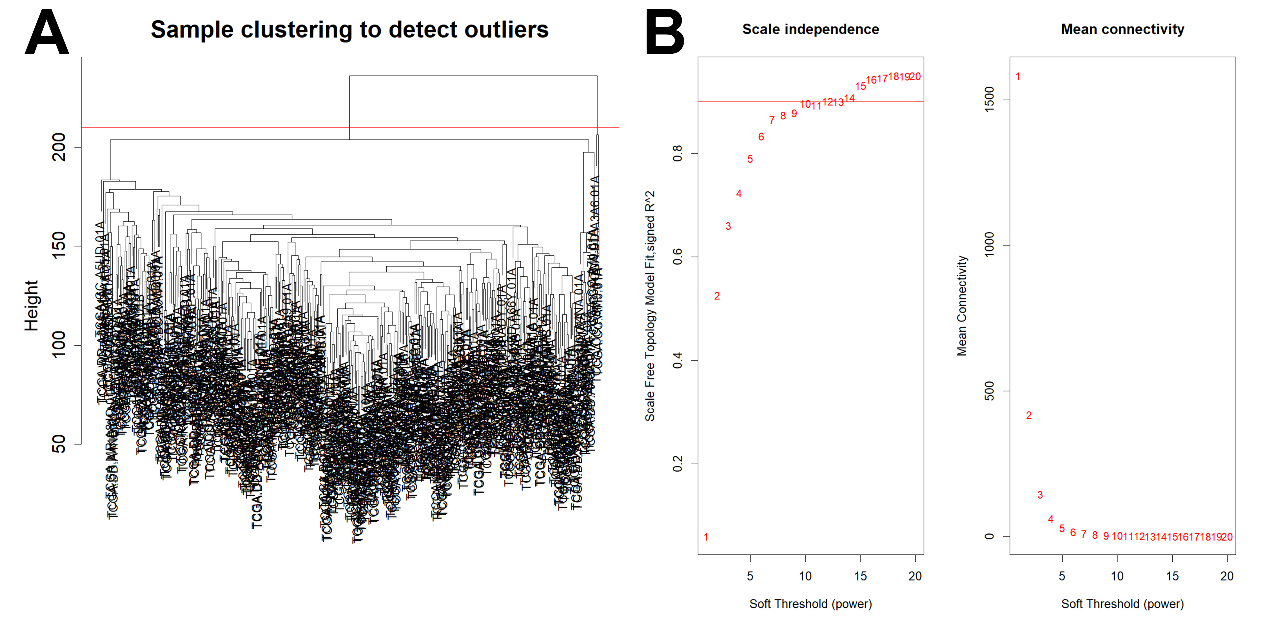


Figure S1 Identify the weighted value β that meets the law of scale-free networks.


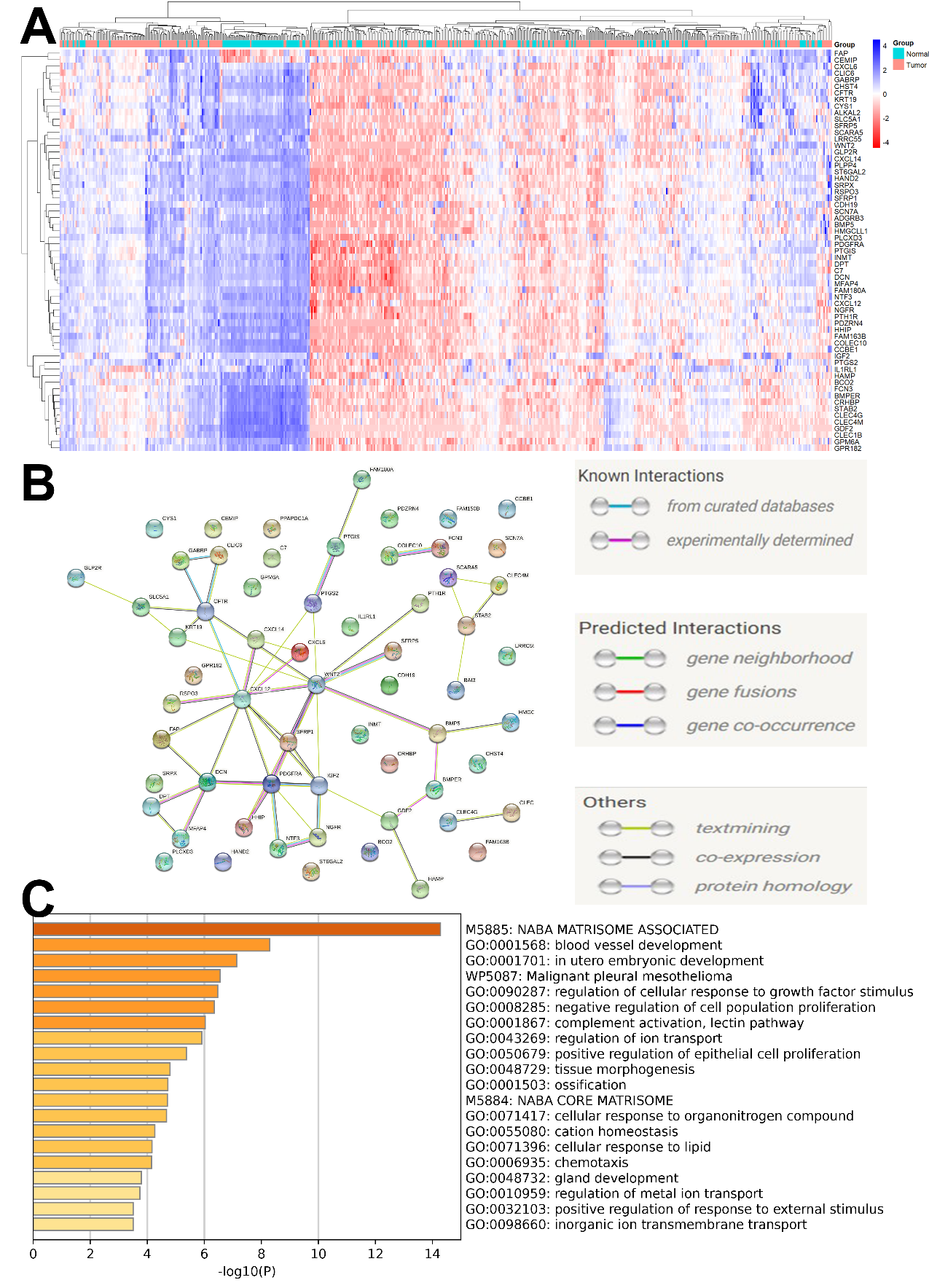


Figure S2 (A) Heatmap of the expression levels of 61 SHRGs in normal and tumor tissues. (B) Protein-protein interaction (PPI) network construction. (C) The main enriched entries for these genes.


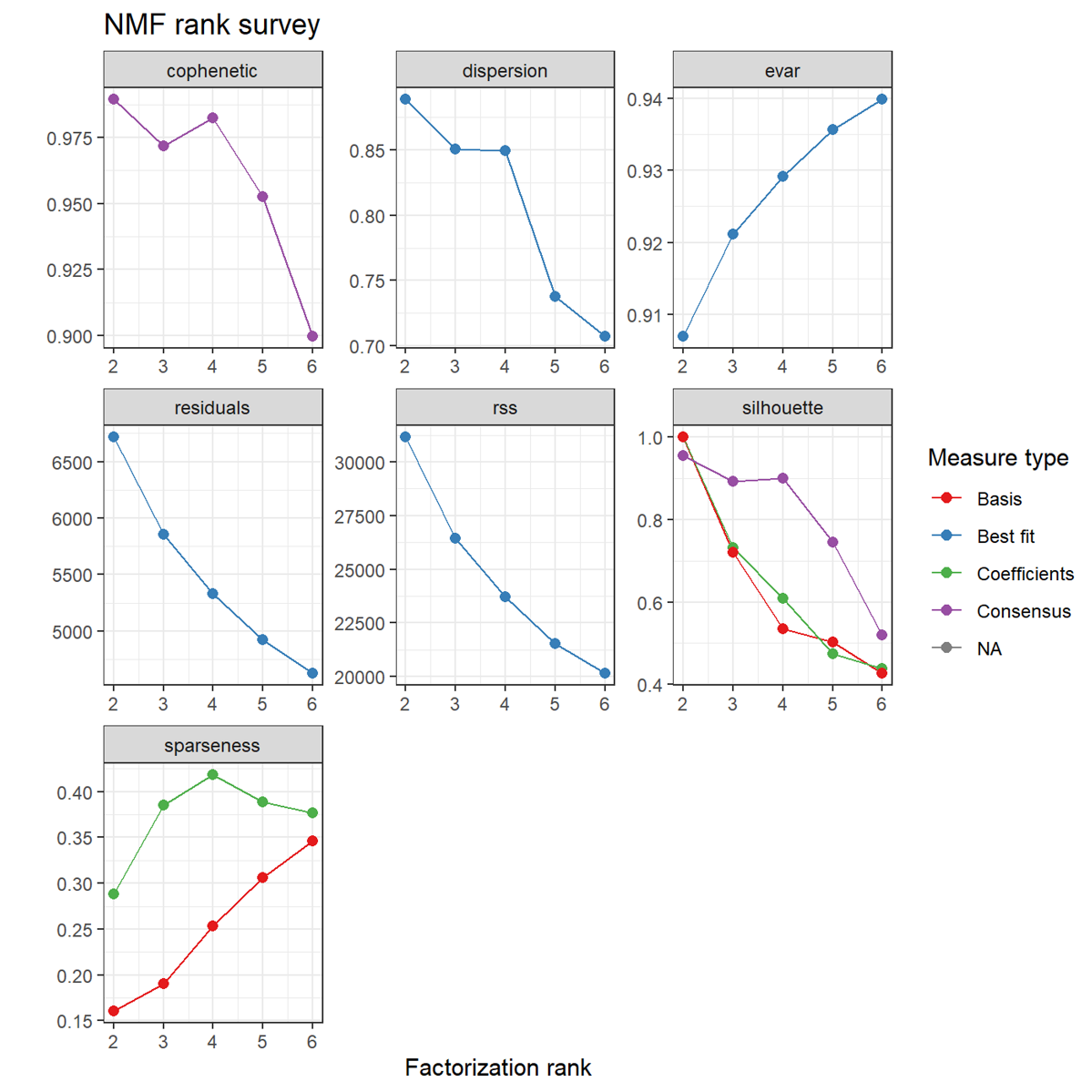


Figure S3 NMF rank survey.


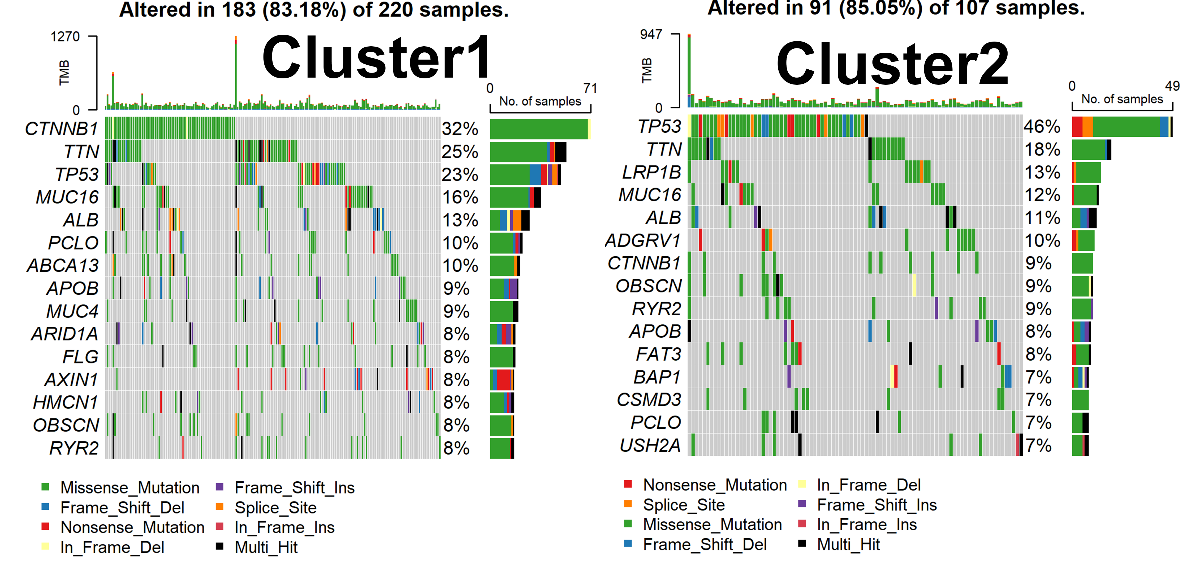


Figure S4 The mutation rates of the top fifteen most significantly mutated genes were significantly different between the Cluster 1 and Cluster 2 subgroups.


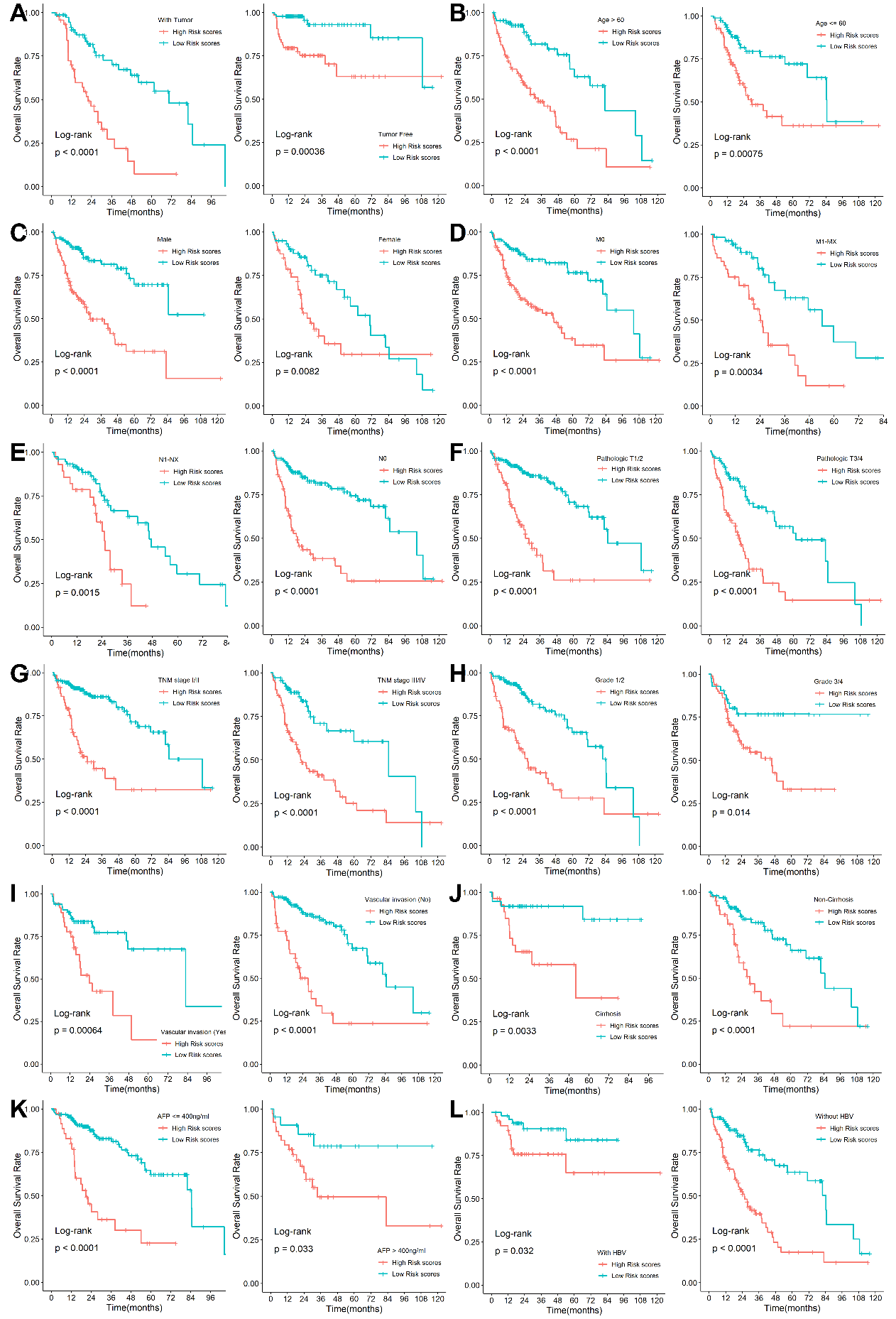


Figure S5 The prognostic model could further differentiate patients with different clinical characteristics.


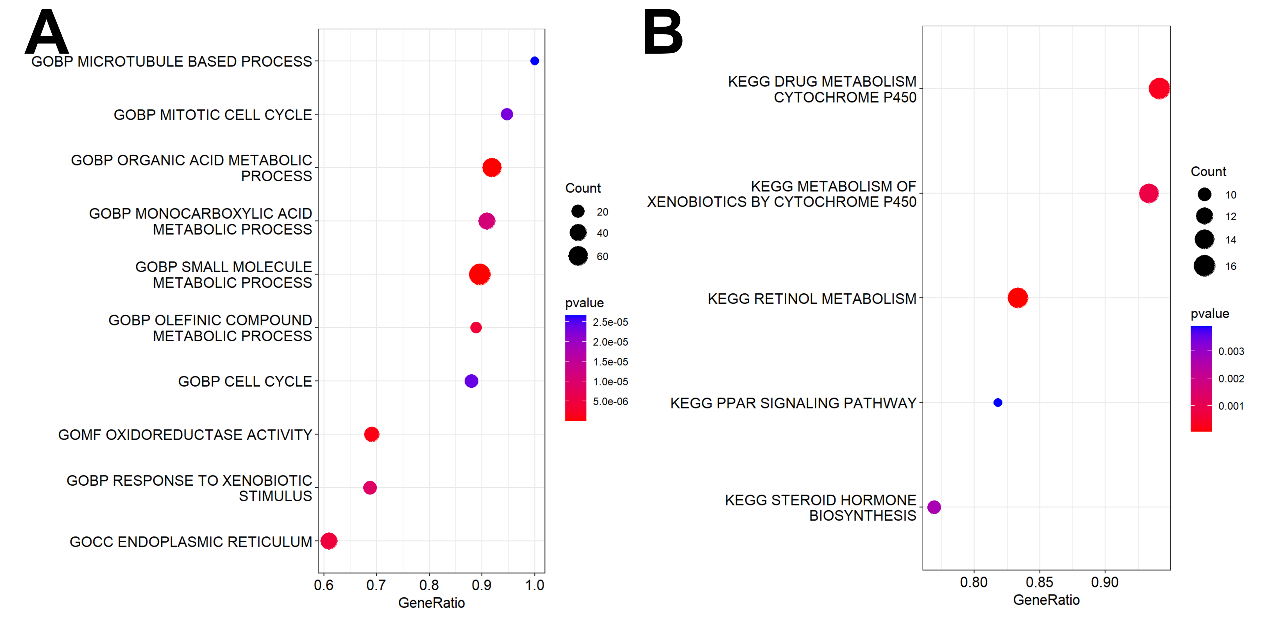


Figure S6 Identification of GO and KEGG enrichment between high- and low-risk scores subgroups.


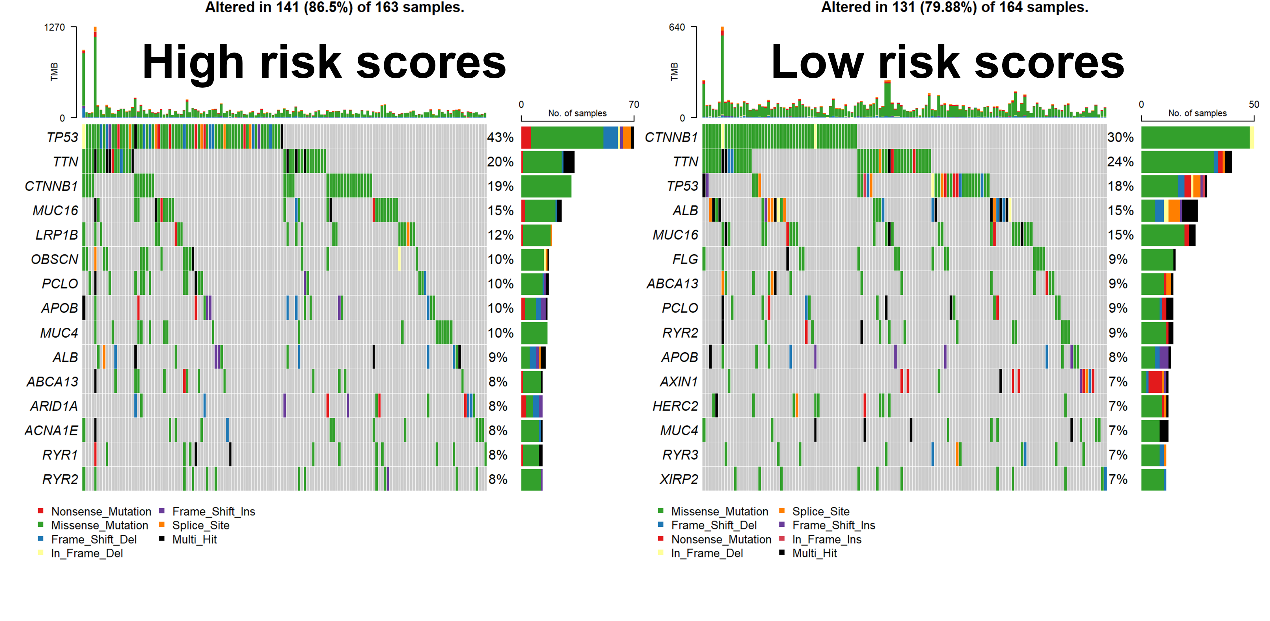


Figure S7 The mutation rates of the top fifteen most significantly mutated genes were significantly different between high- and low-risk scores subgroups.


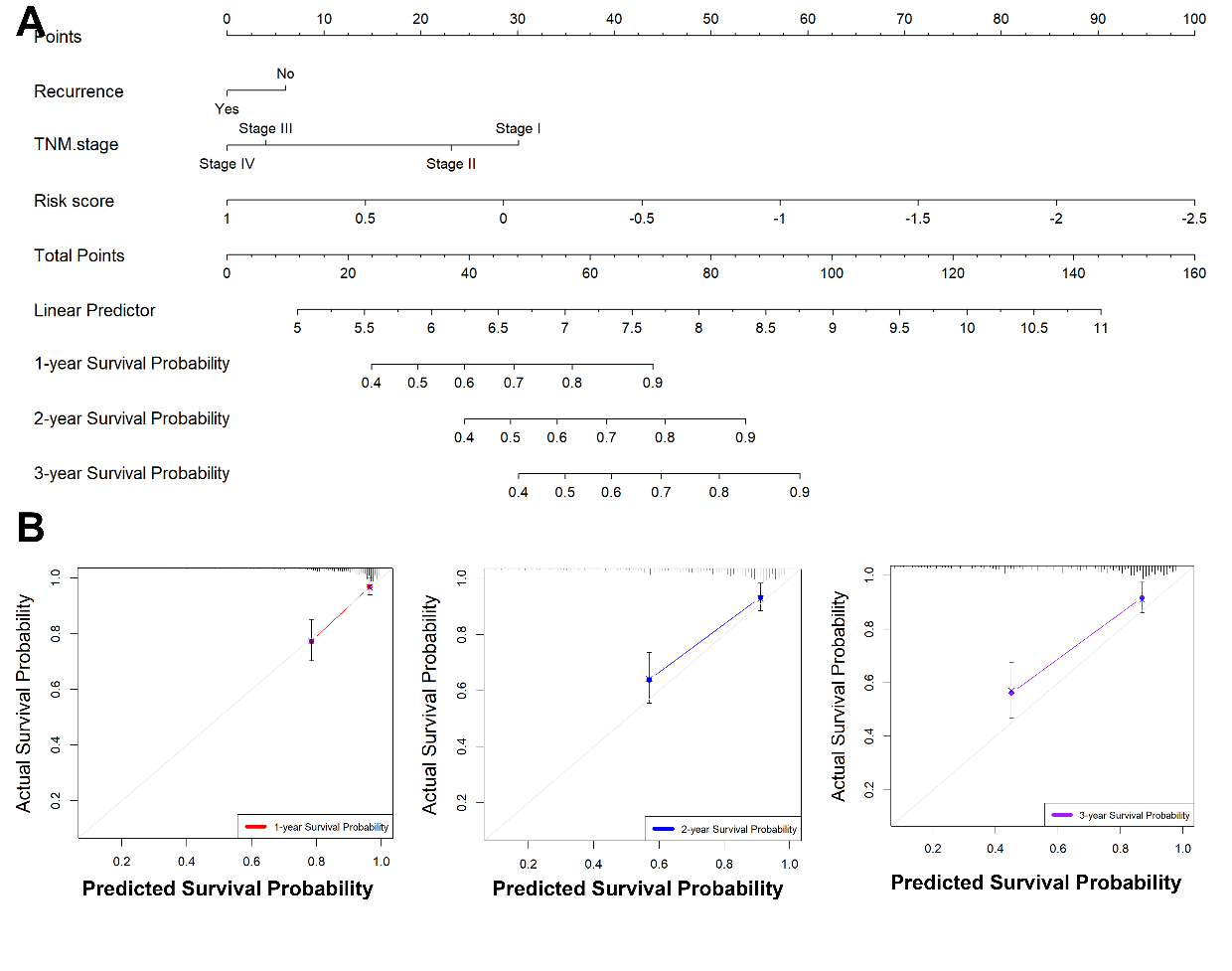


Figure S8 The predictive significance of the prognostic model was verified in the nomogram.


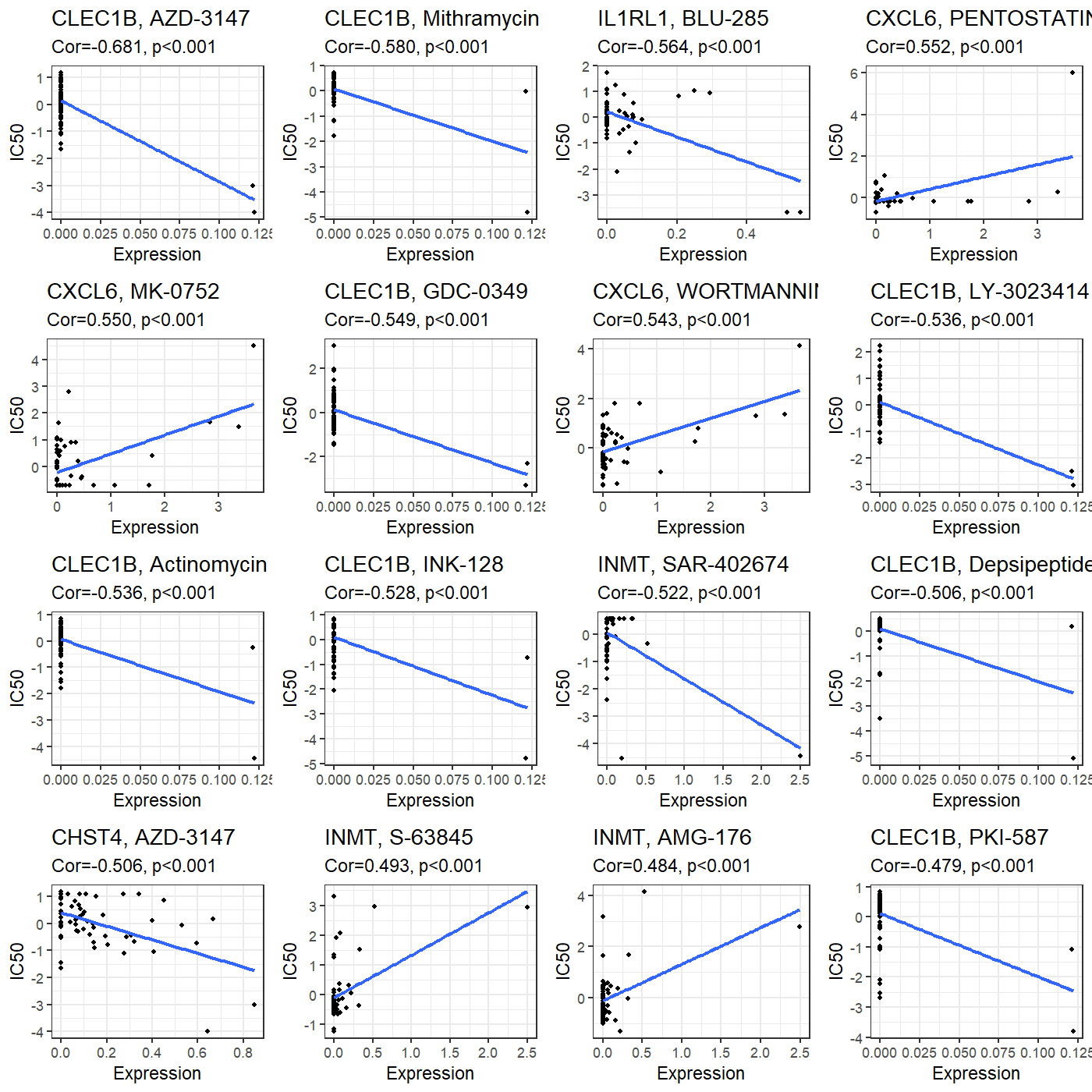


Figure S9 Top 16 most important tumor-sensitive drugs.

Table S1 Clinical characteristics of HCC patients involved in the study

| Characteristics | TCGA cohort  (n=342) | ICGC cohort  (n=227) |
| --- | --- | --- |
| Gender Male | 233 | 61 |
| Female | 109 | 166 |
| Age ≤60 years | 165 | 49 |
| >60 years | 177 | 178 |
| Grade G1/2 | 214 |  |
| G3/4 | 123 |  |
| unknown | 5 |  |
| TNM Stage I/II | 238 | 140 |
| III/IV | 83 | 87 |
| unknown | 21 | 0 |
| Vascular Invasion Yes | 101 |  |
| No | 187 |  |
| unknown | 54 |  |
| Recurrence With tumor | 122 |  |
| Tumor free | 153 |  |
| unknown | 67 |  |
| Cirrhosis With | 65 |  |
| Without | 134 |  |
| unknown | 143 |  |
| HBV or HCV Infection |  |  |
| Yes | 141 |  |
| No | 161 |  |
| unknown | 40 |  |

Table S2 The sequences of the qRT-PCR primers used in this study

| Gene | Forward primer | Reverse primer |
| --- | --- | --- |
| GLP2R | GGAAGTGGGCTCAGTACAAAC | GTCCCGTTACAAAATATGCCAGA |
| C7 | AATGGCTGTACCAAGACTCAGA | GCTGATGCACTGACCTGAAAA |
| IL1RL1 | ATGGGGTTTTGGATCTTAGCAAT | CACGGTGTAACTAGGTTTTCCTT |
| CXCL6 | AGAGCTGCGTTGCACTTGTT | GCAGTTTACCAATCGTTTTGGGG |
| CHST4 | CCTGCTGTTTCTGGTTTCCCA | TGCCCCACAAAAGAAGAGCC |
| GPR182 | CAGTGCCTACCAGTGACCTTG | CACTCAGACAAAGTGTGGTTGA |
| CLEC1B | AGCGCAATTACCTACAAGGTG | CTTCCCATGTTAAGTTGTGCCT |
| INMT | AAGGGGACACGCTGATTGAC | AGTCGGAGAGAGTGATGTCTTG |
| β-ACTIN | CGTGGGCCGCCCTAGGCACCA | TTGGCTTAGGGTTCAGGGGGG |

Table S3 Immune cells with differences in abundance between Cluster1 and Cluster 2 by the four algorithms

| Algorithms | Type of immune cells |
| --- | --- |
| CIBERSORT | memory B cells, resting memory CD4 T cells, follicular helper T cells, Tregs, active NK cells, M0 macrophage, M1 macrophage, M2 macrophage, resting myeloid dendritic cells, resting mast cells, neutrophil, and naive CD4 T cells |
| TIMER | B cells, CD4 T cells, neutrophil cells, macrophage, and myeloid dendritic cells |
| xCELL | memory CD4 T cells, naive CD8 T cells, common lymphoid progenitor, myeloid dendritic cell, cancer-associated fibroblast, macrophage, macrophage M1, neutrophil, Th2 CD 4 T cell, and activated myeloid dendritic cell, and lower abundance levels of central memory CD4 T cells, common myeloid progenitor, endothelial cell, plasma B cell, and Th1 CD 4 T cell |
| MPCcounter | T cells, CD8 T cells, B cell, cancer-associated fibroblast, monocyte, macrophage monocyte, myeloid dendritic cell, neutrophil, and endothelial cell |

Table S4 Immune cells with differences in abundance between high- and low-risk score groups by the four algorithms

| Algorithms | Type of immune cells |
| --- | --- |
| CIBERSORT | activated memory CD4 T cells, follicular helper T cell, Tregs, M0 macrophage, neutrophil, monocyte, and activated mast cells |
| TIMER | CD4 T cells, macrophages, neutrophils, and CD8 T cells |
| xCELL | switched memory B cell, common lymphoid progenitor, common myeloid progenitor, mast cell, NK T cell, Th1 CD4 T cell, Th2 CD4 T cell, naïve CD8 T cell, CD8 T cell, central memory CD8 T cell, endothelial cell, cancer-associated fibroblast, granulocyte monocyte progenitor, hematopoietic stem cell, macrophage, macrophage M2, and plasmacytoid dendritic cell |
| MPCcounter | monocyte, macrophage monocyte, NK cell, and endothelial cell |

Table S5 77 tumor-sensitive drugs targeting tumor cell stemness

| Gene | Drug | correlation coefficient | pvalue |
| --- | --- | --- | --- |
| CLEC1B | AZD-3147 | -0.68127 | 2.88E-09 |
| CLEC1B | Mithramycin | -0.58044 | 1.44E-06 |
| IL1RL1 | BLU-285 | -0.56388 | 3.31E-06 |
| CXCL6 | PENTOSTATIN | 0.551854 | 5.88E-06 |
| CXCL6 | MK-0752 | 0.550435 | 6.29E-06 |
| CLEC1B | GDC-0349 | -0.54939 | 6.60E-06 |
| CXCL6 | WORTMANNIN | 0.543491 | 8.66E-06 |
| CLEC1B | LY-3023414 | -0.53648 | 1.19E-05 |
| CLEC1B | Actinomycin D | -0.53558 | 1.24E-05 |
| CLEC1B | INK-128 | -0.52765 | 1.75E-05 |
| INMT | SAR-402674 | -0.52232 | 2.20E-05 |
| CLEC1B | Depsipeptide | -0.50645 | 4.27E-05 |
| CHST4 | AZD-3147 | -0.50575 | 4.39E-05 |
| INMT | S-63845 | 0.493095 | 7.25E-05 |
| INMT | AMG-176 | 0.484001 | 0.000103 |
| CLEC1B | PKI-587 | -0.47896 | 0.000124 |
| CXCL6 | XAV-939 | 0.462795 | 0.000224 |
| CLEC1B | AZD-8055 | -0.46251 | 0.000226 |
| C7 | S-63845 | 0.454461 | 0.000299 |
| CLEC1B | Dinaciclib | -0.44895 | 0.000362 |
| CLEC1B | A-1210477 | -0.44743 | 0.000381 |
| GPR182 | Imiquimod | 0.441685 | 0.000462 |
| INMT | AZD-5991 | 0.439869 | 0.00049 |
| INMT | Pelitrexol isomer B | -0.43916 | 0.000502 |
| CLEC1B | Hydroxyurea | 0.438292 | 0.000517 |
| CLEC1B | Doxorubicin | -0.43172 | 0.00064 |
| INMT | S-64315 | 0.430816 | 0.000659 |
| IL1RL1 | Ixazomib citrate | -0.42558 | 0.000779 |
| CLEC1B | AZD-2014 | -0.42512 | 0.00079 |
| CXCL6 | Mitotane | 0.422059 | 0.00087 |
| GPR182 | Megestrol acetate | 0.422009 | 0.000871 |
| CLEC1B | MG-132 | -0.42108 | 0.000897 |
| CLEC1B | Imexon | 0.42069 | 0.000908 |
| CXCL6 | Staurosporine | 0.419711 | 0.000936 |
| INMT | Pelitrexol isomer A | -0.41909 | 0.000954 |
| INMT | E-7449 | -0.40554 | 0.00144 |
| CLEC1B | Carfilzomib | -0.40341 | 0.001534 |
| CLEC1B | aldoxorubicin | -0.40184 | 0.001607 |
| INMT | Pelitrexol | -0.40127 | 0.001634 |
| CHST4 | GDC-0349 | -0.39928 | 0.001732 |
| CHST4 | AZD-8055 | -0.39885 | 0.001754 |
| CHST4 | INK-128 | -0.39858 | 0.001768 |
| C7 | Megestrol acetate | 0.393786 | 0.002031 |
| CLEC1B | ICG-001 | -0.39258 | 0.002102 |
| C7 | Isotretinoin | 0.388613 | 0.002353 |
| C7 | S-64315 | 0.387636 | 0.002419 |
| CLEC1B | Cyclophosphamide | 0.386037 | 0.00253 |
| CXCL6 | GSK-2636771 | 0.385725 | 0.002552 |
| CHST4 | GSK-2194069 | 0.385109 | 0.002596 |
| CLEC1B | Sepantronium bromide | -0.38408 | 0.002672 |
| GPR182 | Isotretinoin | 0.38354 | 0.002712 |
| IL1RL1 | Vismodegib | -0.38342 | 0.002722 |
| CHST4 | AZD-2014 | -0.38323 | 0.002736 |
| CHST4 | Linsitinib | 0.380867 | 0.002921 |
| C7 | AZD-5991 | 0.37979 | 0.003009 |
| IL1RL1 | XL-888 | -0.37898 | 0.003076 |
| CHST4 | Hydroxyurea | 0.378704 | 0.0031 |
| CLEC1B | GSK-2194069 | 0.378179 | 0.003145 |
| INMT | MLN-0905 | -0.37807 | 0.003154 |
| CHST4 | RX-3117 | 0.377036 | 0.003244 |
| CHST4 | Fludarabine | 0.376477 | 0.003294 |
| IL1RL1 | MG-132 | -0.37546 | 0.003386 |
| CXCL6 | SW-044248 | 0.375368 | 0.003395 |
| GPR182 | Fluphenazine | 0.37528 | 0.003403 |
| INMT | DACARBAZINE | 0.373283 | 0.003592 |
| CHST4 | LY-3023414 | -0.36769 | 0.00417 |
| CHST4 | JNJ-54302833 | 0.367098 | 0.004237 |
| CXCL6 | 1st Precursor Intermediate to TDP 665759 | 0.366395 | 0.004316 |
| CLEC1B | BIX-01294 | -0.36397 | 0.004599 |
| CLEC1B | JNJ-54302833 | 0.36239 | 0.004794 |
| IL1RL1 | Ixazomib | -0.36162 | 0.00489 |
| GLP2R | DECITABINE | 0.35846 | 0.005307 |
| CLEC1B | PQR-620 | -0.35698 | 0.005512 |
| INMT | RX-3117 | -0.35478 | 0.005831 |
| CLEC1B | Cladribine | 0.350757 | 0.006456 |
| C7 | Veliparib | -0.35072 | 0.006462 |
| CHST4 | Cladribine | 0.350159 | 0.006553 |
| CXCL6 | IWR-1 | 0.349352 | 0.006687 |
| C7 | LOR-253 | -0.34882 | 0.006777 |
| CLEC1B | Homoharringtonine | -0.34838 | 0.006851 |
| CHST4 | UMI-77 | 0.347835 | 0.006945 |
| CLEC1B | AT-7519 | -0.34654 | 0.007172 |
| CLEC1B | Danusertib | -0.3464 | 0.007198 |
| CHST4 | Hydrastinine HCl | -0.34487 | 0.007474 |
| INMT | CEP-9722 | -0.3448 | 0.007488 |
| CHST4 | GSK-2141795 | -0.34435 | 0.00757 |
| IL1RL1 | Bortezomib | -0.3437 | 0.007693 |
| CHST4 | PQR-620 | -0.34336 | 0.007758 |
| C7 | AMG-176 | 0.343293 | 0.00777 |
| CLEC1B | BMS-387032 | -0.34195 | 0.008031 |
| CHST4 | Cordycepin | 0.34186 | 0.008048 |
| CLEC1B | UMI-77 | 0.340937 | 0.008231 |
| CLEC1B | Chelerythrine | 0.340707 | 0.008277 |
| CLEC1B | ONX-0914 | -0.33922 | 0.008582 |
| CLEC1B | TAK-901 | -0.33881 | 0.008667 |
| CXCL6 | Sepantronium bromide | -0.33801 | 0.008837 |
| IL1RL1 | Midostaurin | -0.33441 | 0.009635 |
| CLEC1B | MLN-7243 | -0.33301 | 0.009961 |
